# Conditional Expression of the Small GTPase ArfA Impacts Secretion, Morphology, Growth, and Actin Ring Position in *Aspergillus niger*

**DOI:** 10.3389/fmicb.2018.00878

**Published:** 2018-05-08

**Authors:** Markus R. M. Fiedler, Timothy C. Cairns, Oliver Koch, Christin Kubisch, Vera Meyer

**Affiliations:** Department of Applied and Molecular Microbiology, Institute of Biotechnology, Technische Universität Berlin, Berlin, Germany

**Keywords:** *Aspergillus niger*, tet-on, arf, protein secretion, glucoamylase, GTPase, actin, conditional gene expression

## Abstract

In filamentous fungi, growth and protein secretion occurs predominantly at the tip of long, thread like cells termed hyphae. This requires coordinated regulation of multiple processes, including vesicle trafficking, exocytosis, and endocytosis, which are facilitated by a complex cytoskeletal apparatus. In this study, functional analyses of the small GTPase ArfA from *Aspergillus niger* demonstrate that this protein functionally complements the *Saccharomyces cerevisiae ARF1/2*, and that this protein is essential for *A. niger*. Loss-of-function and gain-of-function analyses demonstrate that titration of *arfA* expression impacts hyphal growth rate, hyphal tip morphology, and protein secretion. Moreover, localization of the endocytic machinery, visualized via fluorescent tagging of the actin ring, was found to be abnormal in ArfA under- and overexpressed conditions. Finally, we provide evidence that the major secreted protein GlaA localizes at septal junctions, indicating that secretion in *A. niger* may occur at these loci, and that this process is likely impacted by *arfA* expression levels. Taken together, our results demonstrate that ArfA fulfills multiple functions in the secretory pathway of *A. niger*.

## Introduction

Members of the fungal kingdom must acquire nutrients from their external environment to survive. An essential prerequisite for this saprophytic lifecycle is the ability to secrete enzymes for the breakdown of extracellular molecules. In filamentous species, secretion also is essential for growth of multicellular, tubular hyphae, whereby membrane components and enzymes necessary for cell wall synthesis are delivered to a continually extending tip.

This high secretion capacity has been harnessed in many biotechnological applications, and fungi such as *Aspergillus niger* are increasingly used as microbial cell factories in the pulp and paper, textile, detergent, beverage, food, agriculture, pharmaceutical, bio-fuel, and chemical industries (Meyer et al., 2016). Despite these utilities, our understanding of the molecular and cellular basis of filamentous growth, hyphal branching, and how these processes are spatially and temporally coupled with secretion, remains incomplete. This has led to constrains for the use of filamentous fungi in biotechnological applications. For example, several attempts to produce industrial relevant recombinant proteins in filamentous fungi led to production rates lower than the capacities published for homologous proteins (Grimm et al., 2005; Fiedler et al., 2013; Meyer et al., 2015). Elsewhere, targeted modification to transcription factors or chaperones for elevated secretion have been only partially successful (van Gemeren et al., 1998; Moralejo et al., 2001; Wiebe et al., 2001; Valkonen et al., 2003; Lombraña et al., 2004). Consequently, rational engineering of production strains with regulated developmental stages for optimal growth, expanded product portfolios, and enhanced secretion during industrial fermentation is currently not possible due to an incomplete understanding of growth and secretion.

With regards to fungal disease, which kills more people per year than malaria, and destroys enough crops to feed ~10% of the world population annually (Cairns et al., 2016), a critical component of virulence for many fungal pathogens is invasive growth of polar hyphae into host tissues. This is often facilitated by secretion of hydrolytic enzymes for nutrient acquisition, or, most notably for plant infecting fungi, secretion of effector molecules which subvert, suppress, or manipulate host immunity to favor infection (Lo Presti et al., 2015). Consequently, a complete understanding of hyphal growth and secretion will enhance our understanding of the molecular basis of disease, and may lead to discovery of novel therapeutic targets.

The widely accepted model postulated by Taheri-Talesh in the model *Aspergillus nidulans* (Taheri-Talesh et al., 2008) states that polarized growth and secretion are coupled at the fungal tip. Vesicles packed with secretory proteins arise from the Golgi by budding (post-Golgi carrier and cargo; Luini et al., 2005), and travel along microtubules and actin filaments with the help of motor proteins to the apical dome, which is marked by cell end markers (Takeshita and Fischer, 2011; Takeshita et al., 2013; Ishitsuka et al., 2015), and enrich in a structure called the Spitzenkörper. Afterwards, they are transported toward the tip where they are tethered to the plasma membrane by a multi protein complex called the exocyst (Riquelme et al., 2014). Subsequent fusion is initiated by the interaction of vesicular-soluble *N*-ethylmaleimide-sensitive-factor attachment protein receptor (v-SNAREs) located within the vesicle membrane and target-SNAREs (t-SNAREs) located at the plasma membrane. Next, the vesicle membrane and membrane bound proteins are incorporated into the plasma membrane, while the vesicular cargo is released into the external environment. While tip elongation proceeds through incorporation of delivered membrane and cell wall assembly via membrane bound enzymes, it is proposed that excess membrane and bound proteins are removed via endocytosis and cycled back to Golgi equivalents or the vacuole, which may ensure tip localization of cell end markers, and, consequently, polarized growth, and secretion (Ishitsuka et al., 2015). Endocytosis occurs at a collar, or ring, of actin patches and associated proteins, such as coronins (Echauri-Espinosa et al., 2012), which is located 1–2 μm behind the hyphal apex in *A. nidulans* (Taheri-Talesh et al., 2008). Although individual actin patches have an average lifespan lasting less than a minute, the position of the actin ring is tightly maintained (Taheri-Talesh et al., 2008). Thus, the position of the actin ring is likely critical for endocytosis, and ultimately filamentous growth. Characterizing key molecular components of this integrated system offers an opportunity to enhance our understanding of secretion and growth. Global gene expression analyses using either microarrays, or more recently, RNA-sequencing, offers an outstanding opportunity to understand growth and secretion at a systems level. In one such effort, Jörgensen et al. analyzed *A. niger* transcriptomes following carbon source dependent enhancement of protein secretion (Jørgensen et al., 2009). In this experiment, maltose and xylose were used as inducing and non-inducing conditions for secretion of the major *A. niger* extracellular protein glucoamylase, respectively. Elevated protein secretion resulted in transcriptional upregulation of over 90 genes encoding proteins which are known or predicted components of the *A. niger* secretory pathway, including glycosylation, protein folding, vesicular transport, and vacuolar sorting. Interestingly, this analysis demonstrated a gene predicted to encode an ADP ribosylation factor (An08g03690, ortholog of *Saccharomyces cerevisiae* Arf1/2) had 30% elevated levels of expression following carbon-dependent enhancement of protein secretion. In addition, one predicted Arf activating protein (An11g02650, ortholog of *S. cerevisiae* Age2) and two predicted Arf guanine nucleotide exchange factors (An07g02190, ortholog of *S. cerevisiae* Sec7; An18g02490, ortholog of *S. cerevisiae* GEA2) were upregulated at a similar subtle level (10–30%). This observation led us hypothesize that (i) the predicted ADP ribosylation factor encoded by An08g03690 is an important regulator of protein secretion in *A. niger* and that (ii) its subtle level of upregulation is critical for ensuring high level secretion.

ADP ribosylation factors of the Arf/Sar family are small GTPase proteins that regulate a diverse range of processes that have been well described in the budding yeast *S. cerevisiae*, including vesicle formation and trafficking, cytoskeletal rearrangements, cell polarity, and budding (Roth, 1999; Lambert et al., 2007; Suda et al., 2018). The family consists of seven members (Arf1, Arf2, Arf3, Arl1, Arl3, Cin4, and Sar1). Arf1 and Arf2 are redundant, and together with Arl1 are major regulators of the secretory pathway, where they control consecutive steps from ER-Golgi, Golgi-ER, Golgi-plasma membrane and endosomes-*trans*-Golgi. Arf1/2 play a critical role in formation of vesicle coats at distinct steps in intracellular vesicle trafficking in the Golgi, specifically formation of COPI vesicles and clathrin coated vesicles at *cis* and *trans* Golgi cisternae, respectively (Roth, 1999; Suda et al., 2018).

Arf proteins, like all small GTPase proteins, cycle between a GTP-bound (active) and GDP-bound (inactive) forms. In their GDP-bound form, small GTPases are distributed within the cytoplasm, but can be targeted toward a specific membrane by their N-terminal myristoylation group binding phospholipids (Franco et al., 1996). Subsequently, GTP exchange factors (GEF) promote the exchange of GDP with GTP, upon which Arf proteins are activated. The GEFs for Arf1 in yeast have been comprehensively identified, and include Gea1/2 and Sec7 (reviewed in Roth, 1999; Suda et al., 2018). GTPase activating proteins (GAP) catalyze the hydrolysis of bound GTP to GDP, thereby inactivating the GTPase, which diffuses back into the cytoplasm. In *S. cerevisiae*, for example, cognate GAPs for Arf1 include Glo3, Age1/2, and Gcs1 (Poon et al., 1996, 1999; Zhang et al., 1998; Suda et al., 2018). In addition to the well characterized role of Arfs in budding yeast, these GTPases have also been well studied in mammals (Gillingham and Munro, 2007), and there is growing interest in the role of these proteins regulating morphology and virulence during disease, for example in the human infecting dimorphic yeast *Candida albicans* and the zygomycete *Mucor circinelloides* (Labbaoui et al., 2017; Patiño-Medina et al., 2017). Despite comprehensive molecular and genetic characterization of Arfs, their cognate GEFs and GAPs, and the regulatory role of these proteins on multiple processes in yeast and mammalian systems, little is known about their role in Aspergilli, and filamentous fungi in general. A single study has demonstrated that an orthologue of *S. cerevisiae* Arf1 and Arf2 localized to Golgi in *A. nidulans* (Lee and Shaw, 2008). Additionally, these authors were unable to recover disruption mutants for *A. nidulans ARF1/2* orthologue, indicating it was essential. Moreover, over-expression of ArfA impacted cell polarity. However, comprehensive functional analyses of this gene and the encoded proteins have not been conducted in an *Aspergillus* spp.

In this work we provide the first functional analysis of ArfA in *A. niger*, and identify a key role of this protein in regulating growth, morphology, and secretion. Conditional expression following replacement of the native *arfA* promoter with the regulatable Tet-on system (Meyer et al., 2011) demonstrated that ArfA is essential. Subsequent loss-of-function and gain-of-function analyses demonstrate that ArfA impacts hyphal growth rate, hyphal tip morphology, and protein secretion. Subcellular localization experiments of fluorescently labeled proteins associated with cytoskeletal elements provide evidence that the position of the endocytic actin ring is impacted by both lowered and elevated levels of *arfA* expression. We also provide new evidence that secretion in *A. niger* may occur at septa.

## Materials and methods

### Strains, growth conditions, and molecular techniques

*A. niger* and *S. cerevisiae* strains used in this study are listed in Table 1. Strains of *A. niger* were grown at 30°C in minimal medium (MM) (Meyer et al., 2010) or complete medium (CM), consisting of minimal medium (MM) supplemented with 1% yeast extract and 0.5% casamino acids. 100 μg/ml of hygromycin, 10 mM uridine, or 10 mM histidine were added to the medium when required. Yeast strains were cultivated at 28°C in YPG (1% yeast extract, 2% peptone, 2% glucose) or YNB (Formedium, UK) supplemented with 2% glucose, 20 mg/l leucine, histidine, uridine, and methionine when needed. FOA counterselection of *A. niger* strains was performed as described earlier (Arentshorst et al., 2012). *S. cerevisiae* counterselection was performed on YNB + FOA plates (Boeke et al., 1984) supplemented with 50 mg/l histidine, 50 mg/l methionine, and 100 mg/l leucine. Strains were grown at 28°C for 3–5 days.

**Table 1:** *A. niger* strains used in this work.

**Name**	**Genotype**	**References**
N402	*cspA1*	Bos et al., 1988
MA70.15	*kusA::amdS, pyrG^−^*	Meyer et al., 2007
FG7	*ΔkusA, pyrG^+^, egfp::sncA-TsncA-AopyrG-TsncA* (derivative of MA70.15)	Kwon et al., 2014
MF9.1	*ΔkusA, pyrG^−^, egfp::sncA, ΔglaA, pyrG^−^* (derivative of MF7.4)	Fiedler, 2017
MF31.2	*ΔkusA, pyrG^+^, egfp::sncA, ΔglaA, PgpdA::glaA_514_-dtomato* (derivative of MF9.1)	This study
MF26.1	*ΔkusA, pyrG^+^, egfp::sncA, ΔglaA, hisB::DR-AopyrG-DR* (derivative of MF9.1)	This study
MF27.1	*ΔkusA, pyrG^−^, egfp::sncA, ΔglaA, pyrG^−^, hisB^−^* (derivative of M26.1)	This study
MF32.6	*ΔkusA, pyrG^−^, egfp::sncA, ΔglaA, hisB^−^, PgpdA::glaA_514_-dtomato* (derivative of M27.1)	This study
FH1.1	*ΔkusA, pyrG^+^, egfp::sncA, ΔglaA, hisB::Tet-on-arfA, PgpdA::glaA_514_-dtomato* (derivative of M32.6)	This study
MF45.5	*ΔkusA, pyrG^+^, egfp::sncA, ΔglaA, hisB::Tet-on-arfA, PgpdA::glaA_514_-dtomato, ΔarfA::hygR* (derivative of FH1.1)	This study
MF47.11	*ΔkusA, pyrG^+^, egfp::sncA, ParfA::arfA::dtomato:hygR* (derivative of FG7)	This study
MA141.1	*ΔkusA, pyrG^+^, PgpdA::glaA::sGFP::HDEL::TtrpC-pyrG* (derivative of MA70.15)	Carvalho et al., 2011
MF48.11	*ΔkusA, pyrG^+^, PgpdA::glaA::sGFP::HDEL::TtrpC-pyrG, ParfA::arfA::dtomato:hygR* (derivative of MA141.1)	This study
Ren1.10	*ΔkusA, pyrG^+^, PgmtA::eYFP::gmtA::TgmtA* (derivative of MA70.15)	Carvalho et al., 2011
MF49.1	*ΔkusA, pyrG^+^, PgmtA::eYFP::gmtA::TgmtA, ParfA::arfA::dtomato:hygR* (derivative of Ren1.10)	This study
MK6.1	*ΔkusA, abpA::ecfp* (derivative of MA70.15)	Kwon et al., 2013
MF46.1	*ΔkusA, pyrG^−^, egfp::sncA, ΔglaA, hisB::Tet-on-arfA, ΔarfA::hygR* (derivative of MF45.5)	This study
MF58.5	*ΔkusA, pyrG^+^, egfp::sncA, ΔglaA, hisB::Tet-on-arfA, ΔarfA::hygR, abpA::ecfp* (derivative of MF46.1)	This study
BY4742	MATα his3Δ1 leu2Δ0 lys2Δ0 ura3Δ0	Baker Brachmann et al., 1998
3890	MATa, his3Δ1, leu2Δ0, met15Δ0, ura3Δ0, ydl192w::kanMX	Winzeler et al., 1999
MF52.2	MATa, his3Δ1, leu2Δ0, met15Δ0, ydl192w::kanMX, pRS416::ARF1::URA3 (derivative of 3890)	This study
MF59.1	MATa, his3Δ1, leu2Δ0, ydl192w::kanMX, pRS416::ARF1::URA3, arf2::MET15 (derivative of MF52.2)	This study
MF61.1	MATa, his3Δ1, leu2Δ0, ydl192w::kanMX, pRS416::ARF1::URA3, arf2::MET15, pRS315PGI-ARF1 (derivative of MF59.1)	This study
MF62.1	MATa, his3Δ1, leu2Δ0, ydl192w::kanMX, pRS416::ARF1::URA3, arf2::MET15, pRS315PGI-*arfA* (derivative of MF59.1)	This study
CK5.1	MATa, his3Δ1, leu2Δ0, ydl192w::kanMX, pRS416::ARF1::URA3, arf2::MET15, pRS315PGI (derivative of MF59.1)	This study

All molecular techniques were performed according to standard procedures (Green and Sambrook, 2012). Plasmids were constructed using Gibson assembly (Gibson, 2011) and the transformation, genomic DNA extraction and Southern blot were performed as described elsewhere (Meyer et al., 2010).

Protein production in shake flasks was performed as follows: 5 × 10^6^ conidia/ml were inoculated in 20 ml MM medium supplemented with 5% glucose and different concentrations of doxycycline in 100 ml Erlenmeyer flasks, and cultivated at 30°C and 250 rpm on a horizontal shaker for 72 h. One milliliter samples for microscopic analysis were taken, the biomass was harvested by filtration, washed once with physiological salt solution and supernatants were collected for further analysis.

### Construction of a *PgpdA::glaA_514_::dtomato* construct

*PgpdA::glaA*_514_*::dtomato* expression construct (pMF30.1) was constructed by amplification of *glaA*_514_ and *dtomato* via PCR (Supplementary Table S1). For dtomato, plasmid pBN018-60-61 (van Munster et al., 2013) was used as template and the short version of the glucoamylase gene *glaA*_514_ was amplified from genomic DNA of N402. Both fragments were cloned into the EcoRI and PmeI opened plasmid pVG2.2 (Meyer et al., 2011) via Gibson assembly (Gibson, 2011).

### Construction of a *hisB* disruption and *Tet-On::arfA::hisB* expression construct

The construction of the *hisB* disruption construct is described elsewhere (Fiedler et al., 2017). The *Tet-On::arfA::hisB* expression plasmid (pFH1.3) was created by inserting *arfA* amplified via PCR (Supplementary Table S1) from the genomic DNA of N402 into the unique PmeI restriction site of *Tet-On::hisB* (pTG1.2 Fiedler et al., 2017) via Gibson assembly.

### Construction of a *arfA::hygR* deletion cassette

The 5′ and 3′ regions of *arfA* and the hygromycin resistance gene were amplified in a bipartite approach as published recently (Arentshorst et al., 2015) using primers listed in Supplementary Table S1 and cloned into pJET1.2 (Thermo, USA) via Gibson assembly giving rise to bipartite containing plasmids pCK1.1 (*ParfA-hygR*) and pCK2.6 (*hygR::TarfA*), respectively. Both plasmids were linearized with DraI giving rise to two bipartites with a length of 3.1 kb each.

### Construction of a *arfA::dtomato::hygR::TarfA* cassette

C-terminal ArfA labeling was chosen because of an N-terminal myristoylation site which was shown to be essential for proper localization (Liu et al., 2009). The promoter sequence of *arfA, dtomato-TtrpC* and the hygromycin resistance gene were amplified using primers listed in Supplementary Table S1 from genomic DNA of N402, pMF30.1, or pAN7.1(Punt et al., 1987) using PCR and ligated into the XhoI and NdeI opened plasmid pCK2.6 via Gibson assembly giving rise to pMF44.6.

### Microscopy

Different cultivation techniques were applied for microscopy. Cultivation for cLSM microscopy of *A. niger* was performed as described recently (Kwon et al., 2014). In brief, spores were spotted in MM plates, supplemented with different concentrations of doxycycline when needed and incubated at 22°C for 2 days, following cutting out of the colony and placing it upside down into a glass bottom petri dish (Kwon et al., 2014). Liquid MM medium (supplemented with the same concentration of doxycycline which was present in the MM plate) was added and cells were incubated at 22°C until they resumed growth. Fluorescence and DIC images were taken using an inverted TCS SP8 (Leica, Germany). For fluorescence microscopy, cultivation was performed as described earlier (Fiedler et al., 2014). In brief, cover slides were placed on the bottom of a Petri dish containing 5 ml MM medium (supplemented with 100 mM MES pH6.5 and different concentrations of doxycycline when needed), inoculated with 1 × 10^5^ spores/ml and cultivated at 28°C for 12 h. Images were acquired using a DMI6000 fluorescence microscope (Leica, Germany).

### Growth-plate assays

Defined spore titers of *A. niger* strains were spotted on MM medium supplemented with different additives and varying doxycycline concentrations in biological triplicate and incubated at 30°C for 3 days, after which representative images were captured.

### Glucoamylase analysis

Western analysis was performed according to standard procedures (Green and Sambrook, 2012) and as described earlier (Punt et al., 1998). Briefly, a monoclonal anti-glucoamylase antibody produced in mouse (kindly provided by Prof. Peter Punt, University of Leiden) was used as a primary antibody. The primary antibody was detected with an anti-mouse-HRP conjugated antibody (Agilent Technologies, USA). Primary and secondary antibody incubations were performed in PBS-T (137 mM NaCl, 2.7 mM KCl, 1 mM Na_2_HPO_4_, 0.2 mM KH_2_PO_4_, 0.1% Tween-20) supplemented with 5% dry milk. The primary antibody incubation was performed at 4°C for 16 h, after which the blot was incubated with the secondary antibody at room temperature for 1 h. Chemiluminescence reaction was performed by using an ECL Prime Western Blotting Detection Kit (GE Healthcare). Band intensities were analyzed using the open source program ImageJ and the signal intensity was normalized against the corresponding biomass and subsequently against the signal intensity of the corresponding control strain.

### Construction of the *S. cerevisiae* complementation strains

To construct *S. cerevisiae* strains which were used for complementation analysis, we followed an approach which was published earlier (Takeuchi et al., 2002). In brief, the *S. cerevisiae ydl192w::kanMX* (3890) strain from the *Saccharomyces* Genome Deletion Project (Winzeler et al., 1999) lacking a functional copy of *ARF1* was transformed with the ectopically replicating plasmid pMF46.3 derived from pRS416 (purchased from Stratagene) giving rise to MF52.2. pMF46.3 carries the full length *ARF1* gene flanked by 471 bp of the 5′ and 833 bp of the 3′ region of *ARF1* amplified from the genome of BY4742 using primers listed in Supplementary Table S1 ligated into pRS416 using unique BamHI and EcoRI restriction sites. Subsequently, an ARF2::MET15 deletion cassette was amplified from the genome of BY4742 using primers listed in Supplementary Table S1 and transformed into MF52.2 to create MF59.1. Finally GPD driven expression plasmids for *ARF1* (pMF48.1) or *arfA* (pMF49.1) were constructed via inserting the respective genes amplified via PCR from the genome of BY4742 and N402, respectively into a unique BamHI restriction site of the ectopically replicating expression plasmid pRS315-PGI (kindly provided by Dr. T. H. Chang, Ohio State University, Weaver et al., 1997) carrying LEU2 as selection marker and subsequent transformation into MF59.1 to generate MF61.1 (*ARF1*), MF62.1 (*arfA*), or CK5.1 (plasmid control).

## Results

### The *A. niger* ArfA encoding gene functionally complements *S. cerevisiae ARF1/2*

**Figure 1:**
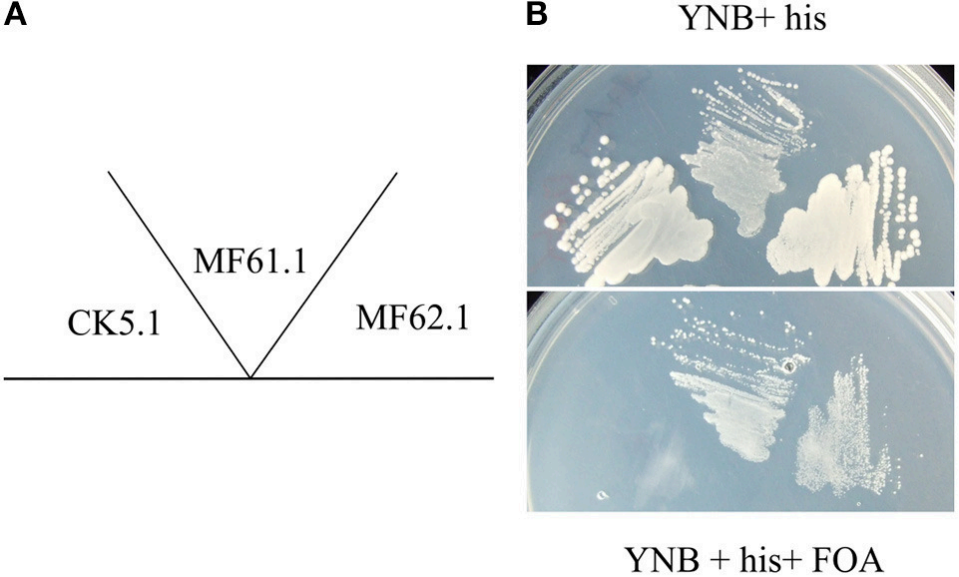
*A. niger* ArfA functionally complements S. cerevisiae Arf1/2. The *S. cerevisiae ARF1/2* double null, containing an FOA counterselectable *ARF1* expression plasmid, was transformed with vectors expressing the native *S. cerevisiae ARF1* (MF61.1), *A. niger arfA* (MF62.1), or empty vector control (CK5.1). These strains were grown on agar plates as depicted schematically **(A)**. Media consisted of yeast nitrogen base (YNB) supplemented with 10 mM histidine (his, upper panel), which was supplemented with FOA (lower panel). CK5.1 was unable to grow following counterselection, whereas both isolates expressing the *S. cerevisiae ARF1* or *A. niger arfA* demonstrated comparable growth, **(B)** indicating *A. niger arfA* functionally complements *ARF1/2*.

Microarray analyses of *A. niger* gene expression during carbon source dependent upregulation of secretion has identified transcriptional deployment of numerous genes, including those predicted to function in protein folding, *N*-glycosylation, vesicle packaging, and ER to Golgi transport (Jørgensen et al., 2009). Interestingly, these analyses also identified upregulated expression of gene An08g03690, which is predicted to encode an Arf protein, during elevated secretion. Subsequent BLAST analyses of the encoded amino acid sequence revealed An08g03690 has sequence homology of 76.1% when compared with *S. cerevisiae* Arf1 (Supplementary Table S2). Further BLAST searches of the An08g03690 protein sequence revealed 97.8% sequence homology to the *A. nidulans* ArfA (Lee and Shaw, 2008, data not shown). We consequently named the protein encoded by the An08g03690 gene in *A. niger* ArfA. In order to assess whether the An08g03690 gene encodes an ADP ribosylation factor, we conducted a *S. cerevisiae* complementation assay comparable to those previously described (Takeuchi et al., 2002). We firstly generated *S. cerevisiae* strain MF59.1, which expresses an episomal plasmid constitutively expressing a functional *ARF1* gene that can be removed via 5-fluororotic acid (FOA) counterselection (Figure 1). This isolate is also deleted for genes YDL192W and YDL137W, which encode proteins Arf1 and Arf2, respectively. Given that deletion of both paralogues is lethal in *S. cerevisiae*, FOA counterselection in isolate MF59.1 must be complemented with supply of a functional ARF for cell survival. Next, an expression construct containing the *A. niger arfA* gene (An08g03690) was transformed into MF59.1, giving isolate MF62.1. Additionally, plasmids encoding the endogenous *S. cerevisiae ARF1*, and an unmodified vector control, were also introduced to MF59.1, to give strains MF61.1 and CK5.1, respectively. These isolates were used as positive and negative controls for functional ARF complementation in the subsequent assay. All three strains were able to grow on YNB supplemented with histidine (Figure 1). Plating the three strains on YNB supplemented with histidine and FOA revealed no growth for the negative control (CK5.1), but normal growth for the *A. niger arfA* (MF62.1) and endogenous ARF1 (MF61.1) complemented strains (Figure 1). This assay confirmed that the *A. niger arfA* is able to complement loss of *ARF1/2* in *S. cerevisiae* and also provides indirect evidence that the protein encoded by this gene functions is an ADP ribosylation factor.

### Gene functional analysis demonstrates *arfA* is essential in *A. niger*, and is required for normal growth and resistance to secretion stress

**Figure 2:**
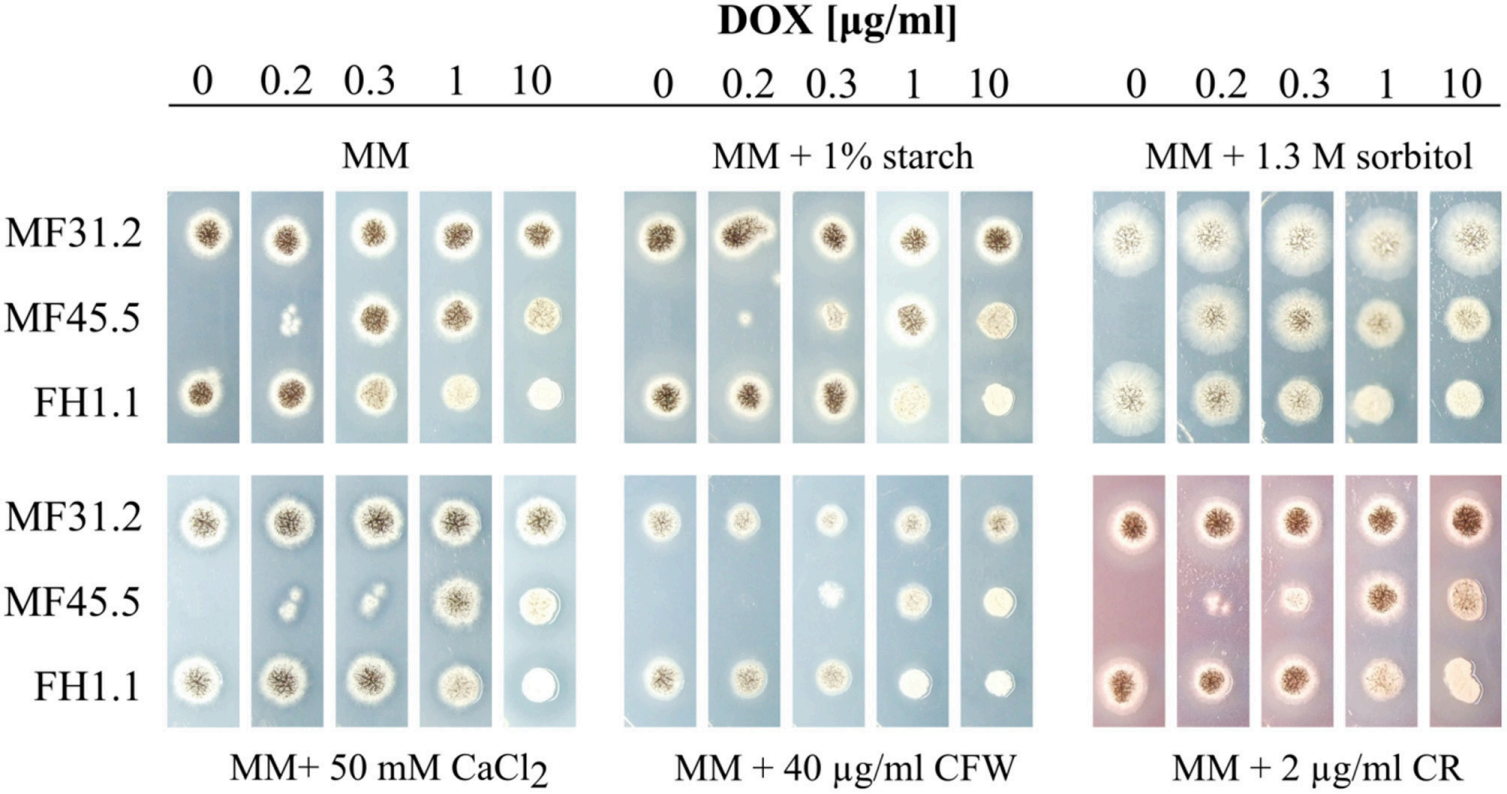
ArfA is essential in *A. niger*, and modifications to *arfA* expression cause sensitivity to various secretory stressors. An ArfA gain-of-function strain FH1.1 was constructed by placing the *arfA* gene under control of the doxycycline inducible Tet-on system. For loss-of-function analyses, strain MF45.5 was generated by deletion of the native *arfA* gene in isolate FH1.1. All growth was compared in biological triplicate to the isogenic progenitor strain (MF31.2). 5 × 10^3^ spores were inoculated on MM supplemented with various abiotic stressors that cause perturbation of the fungal secretory pathway or cell wall. Plates were incubated at 30°C for 3 days, after which representative images of colony growth were captured. Various concentrations of doxycycline (DOX) enabled titration of *arfA* expression in conditional expression mutants. Perturbation with congo red (CR) and calcofluor white (CFW) are indicated. Complete lack of growth in MF45.5 lacking DOX indicated ArfA is essential in *A. niger*.

As deletion of both paralogues *ARF1/2* in *S. cerevisiae*, and *arfA* in *A. nidulans* is lethal (Stearns et al., 1990; Lee and Shaw, 2008), we reasoned that the gene might be essential in *A. niger*. In support of this hypothesis, 4 independent transformations did not result in successful purification of a homokaryotic deletion strain. Indeed, only heterokaryons were rescued from primary transformation plates, and subcultured spores were unable to grow on MM supplemented with hygromycin (data not shown). In order to assess the *arfA* loss-of-function phenotype of in *A. niger*, we placed the *arfA* gene under control of the doxycycline inducible Tet-on system (Meyer et al., 2011) integrated into the *hisB* locus (Fiedler et al., 2017), to generate isolate FH1.1. The Tet-on conditional expression system is titratable in *A. niger*, and enables accurate modifications of gene expression by addition of the highly stable tetracycline derivative doxycycline to growth media (Meyer et al., 2011; Helmschrott et al., 2013; Wanka et al., 2016). For isolate FH1.1, which contains the endogenous *arfA* gene, any addition of doxycycline to growth media results in *arfA* overexpression. For loss-of-function analyses, strain MF45.5 was generated by deletion of the native *arfA* gene in isolate FH1.1. In order to assess protein secretion in subsequent microscopic and Western blot experiments, both isolates also constitutively expressed a C-terminal dtomato tagged glucoamylase gene *glaA*, under control of the constitutive *A. niger* glyceraldehyde-3-phosphate dehydrogenase promoter at the *pyrG* locus (Table 1). Consequently, for growth assays, we used isolate MF31.2 as a control, which also constitutively expresses a C-terminally dtomato tagged *glaA*, but has no modifications to *arfA* expression (Table 1). Titration of *arfA* gene expression by addition of various concentrations of doxycycline to growth media allowed us thus to study loss-of-function and gain-of-function phenotypes in the same isolate. As shown in Figure 2, the doxycycline controllable *arfA* expression strain MF45.5 was unable to grow on plates lacking doxycycline, while addition of 0.2 μg/ml of doxycycline led to an intermediate phenotype showing reduced growth and sporulation in comparison to the wildtype progenitor strain. These data demonstrate that *arfA* is indeed essential in *A. niger*. With increased doxycycline concentration (0.3 and 1 μg/ml), wildtype-like growth phenotype was restored in MF45.5. Growth and sporulation of *A. niger*, however, were significantly reduced when *arfA* was overexpressed at 10 μg/ml doxycycline for both *arfA* expression strains (MF45.5 and FH1.1), demonstrating not only a clear overexpression phenotype but also suggesting that native *arfA* expression itself is under delicate control to ensure proper growth. In order to test whether the observed growth defects in *arfA* conditional expression isolates were due to mis-regulation of the secretory pathway, we conducted phenotypic screens of these mutants under conditions known to affect or perturb fungal secretion, including growth on starch as a sole carbon source, which requires secretion of amylolytic proteins for external substrate digestion. Growth of isolate MF45.5 was strongly impaired compared to wild-type when grown on starch media supplemented with 0.2–0.3 μg/ml doxycycline, suggesting a defect in secretion (Figure 2). Interestingly, overexpression of *arfA* also resulted in sensitivity to secretion stress in isolate MF45.5. We also tested growth on MM supplemented with calcium, which is a central regulator of the secretory pathway of filamentous fungi and yeasts (Sambrook, 1990; Lew, 2011; Liu, 2012; Figure 2). Strain FH1.1, which has an endogenous *arfA* copy and a Tet-on regulated allele did not display sensitivity to secretion stress at low doxycycline concentrations (0–0.3 μg/ml), yet at higher concentrations (1–10 μg/ml) demonstrated reduced growth relative to the control (Figure 2), further suggesting that overexpression of *arfA* results in sensitivity to secretion stress.

Given that the secretory pathway is essential for transporting cell wall synthesizing enzymes and material toward apical regions during hyphal growth, we reasoned that loss-of-function and gain-of-function *arfA* mutants would also be sensitive to cell wall interfering compounds such as calcofluor white (CFW) and congo red (CR), which are known inhibitors of chitin and glucan assembly, respectively (Roncero and Durán, 1985; Ram and Klis, 2006; He et al., 2014). Growth of strain MF45.5 was indeed stronger impaired than the respective control strains FH1.1. and MF31.1 on plates containing CFW and CR (Figure 2), strongly supporting the hypothesis of defective delivery of cell wall components in *arfA* loss and gain-of-function strains. We finally challenged the strains with sorbitol, which causes osmotic stress at both the plasma membrane and cell wall. Growth in strain MF45.5 under decreased *arfA* expression was comparable to that of the progenitor strain, and minor defects in sporulation were observed under native or overexpression conditions (Figure 2). Taken together, these data suggest that ArfA plays essential roles regulating the secretion pathway in *A. niger*.

### Titration of *arfA* expression impacts *A. niger* secretion and colony macromorphology in submerged culture

**Figure 3:**
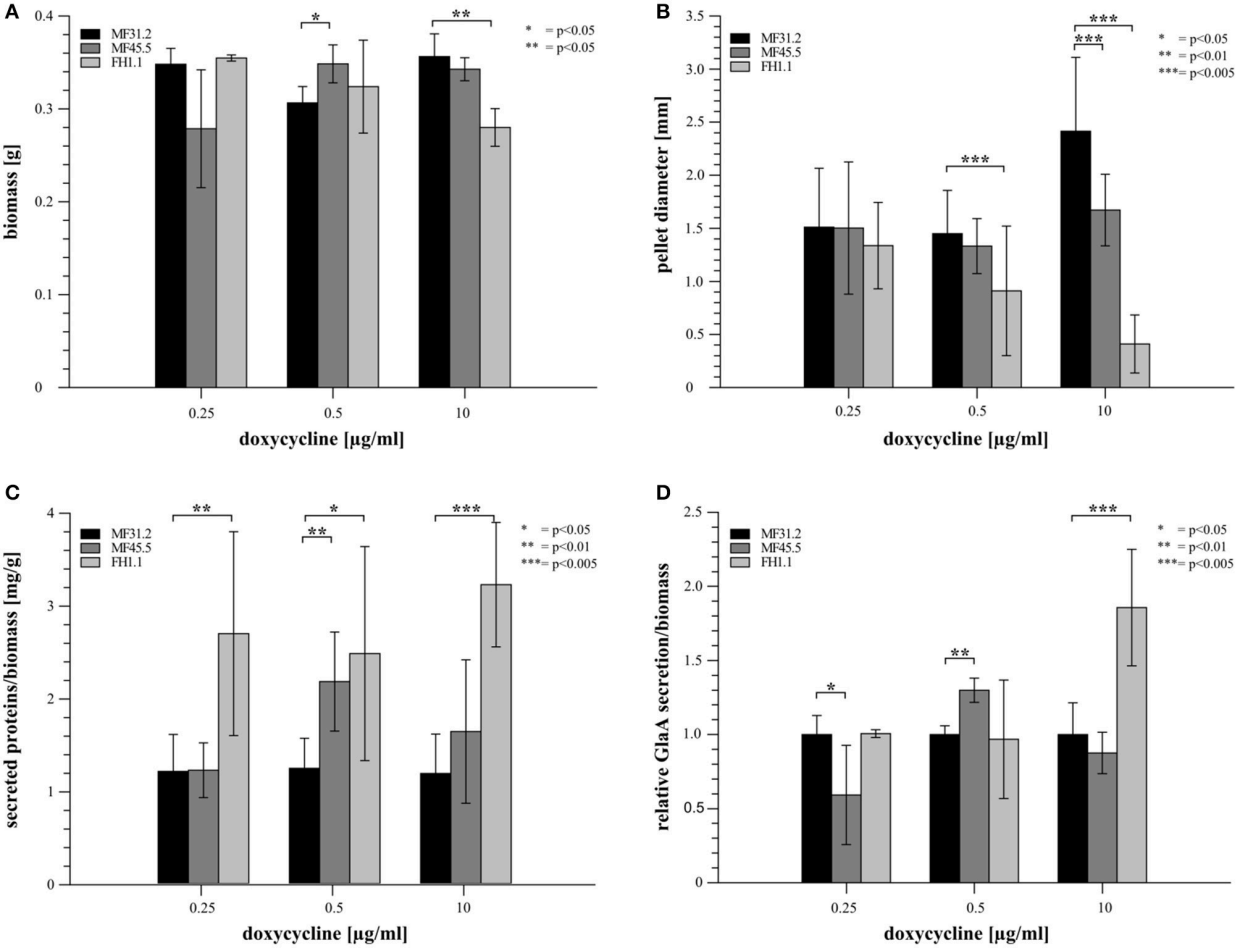
Modification of *arfA* expression impacts colony macromorphology and protein secretion in submerged cultures. 20 ml of MM supplemented with 5% glucose and various doxycycline concentrations (0.25, 0.5, and 10 μg/ml) were inoculated with a final concentration of 5 × 10^6^ spores/ml and incubated at 30°C for 72 h. Each cultivation was performed in biological triplicates. Cultures were analyzed for their dry biomass production 72 h after cultivation **(A)** and pellet diameter **(B)**. The amount of secreted proteins/g dry biomass **(C)** was analyzed using a Bradford assay. The amount of secreted GlaA **(D)** was monitored via Western analysis and normalized firstly against the dry biomass for each corresponding strain/condition. A second normalization was conducted where GlaA was reported relative to the control strain MF31.2 at each respective condition. Students *t*-test was used for significance determination and *p*-values are reported.

We hypothesized that *arfA* downregulation and overexpression would affect the total protein secretion capacity of *A. niger*. We therefore measured secretion of fluorescently tagged glucoamylase GlaA, one of the major secreted proteins in *A. niger*, in addition to total extracellular protein, after 72 h growth in shake flask culture (Figure 3 and Supplementary Figure S1). We also measured biomass and pellet diameter to assess macromorphological changes following *arfA* conditional expression (Figure 3 and Supplementary Figure S2). We detected minor but statistically significant modifications to biomass in *arfA* loss- and gain-of-function mutants at 0.5 and 10 μg/ml doxycycline respectively (Figure 3A). Interestingly, *arfA* overexpression resulted in reduced pellet diameter (Figure 3B), which may be favorable for industrial fermentation applications (Wucherpfennig et al., 2012). Additionally, total protein secretion was increased in ArfA overexpression isolate FH1.1 under all conditions (Figure 3C). This, however, cannot be explained exclusively by changes in pellet diameter, as secretion was also elevated in FH1.1 at 0.25 μg/ml doxycycline, when *A. niger* macromorphology was comparable to control strains (Figure 3B). Extracellular GlaA (Figure 3D) was elevated in FH1.1 (10 μg/ml doxycycline) suggesting that higher levels of *arfA* expression results in increased secretion of this protein (Figure 3D). It should be noted that in contrast to isolate FH1.1, strain MF45.5 demonstrated increased GlaA secretion exclusively at 0.5 μg/ml doxycycline, which could be due to changes in pellet diameter between these isolates at various doxycycline concentrations (Figure 3B).

With regards to analyses of *arfA* downregulation using 0.25 μg/ml doxycycline in mutant MF45.5, colony macromorphology was comparable, yet GlaA production was reduced relative to the MF32.1 control (Figure 3D). Interestingly, total protein secretion in MF45.5 during downregulation of *arfA* was also comparable (Figure 3C). These data suggest that lower *arfA* levels might impact a GlaA-specific component of the secretory system, but does not detectably impact the total secretion capacity in *A. niger*. In summary, our data indicate that (i) overexpression of the ArfA encoding gene elevates secretion of total protein and GlaA, while concomitantly reducing pellet diameter and (ii) lower ArfA levels causes a reduced secretion of GlaA but not total protein.

### Reduced *arfA* expression results in hyphal bursting at intercalary regions

**Figure 4:**
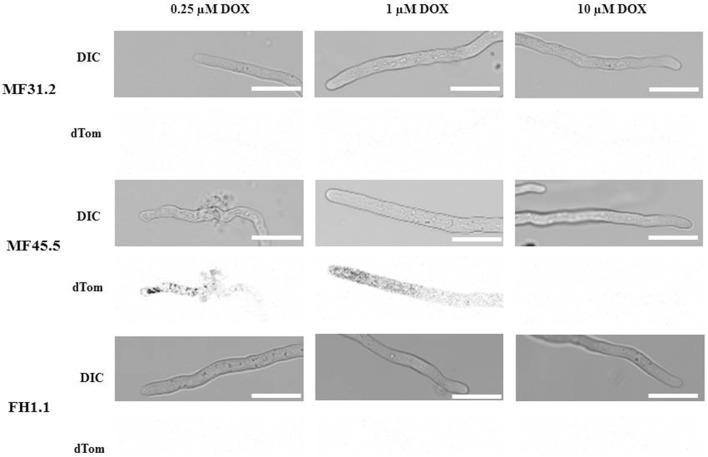
Decreased *arfA* expression levels impact GlaA secretion and cause intercalary hyphal rupturing. Strains MF31.2, MF45.5, and FH1.1 were cultivated on MM supplemented with concentrations of doxycycline as indicated at 22°C for 2 days and analyzed via confocal microscopy. Representative pictures are shown for doxycycline concentrations of 0.25, 1, and 10 μg/ml for DIC images **(Upper)**, and GlaA-dtomato **(Lower)**. GlaA remained intracellular under loss-of-function conditions. Reduced *arfA* expression in isolate MF45.5 also led to significant defects in hyphal morphology and intercalary hyphal bursting. Scale bar is 10 μm.

**Figure 5:**
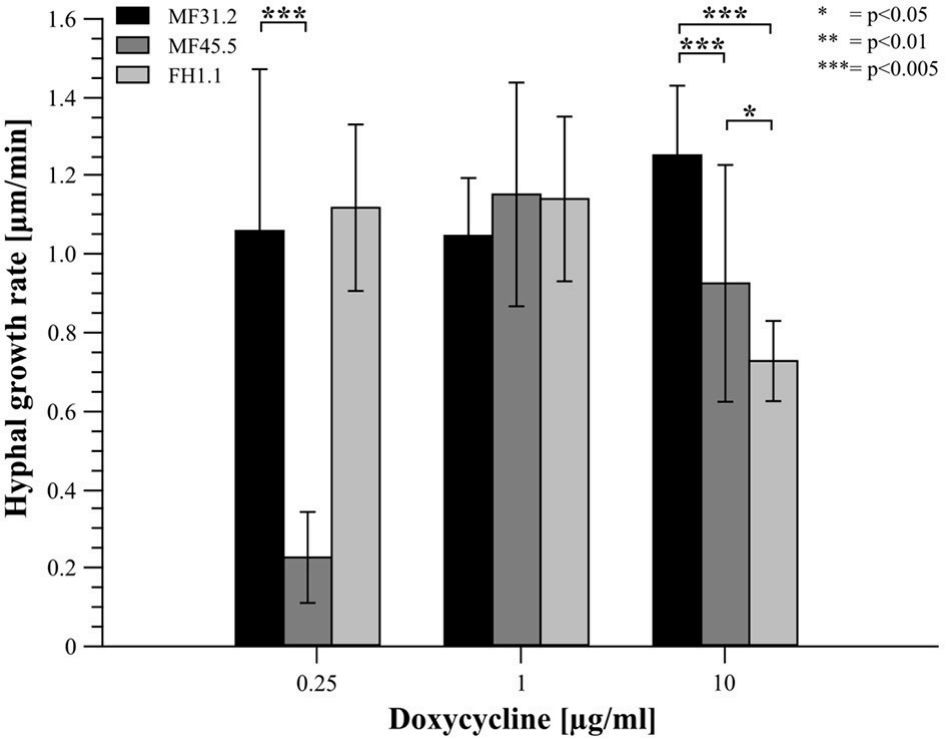
Elevated and reduced *arfA* expression decreases the growth rate of *A. niger* hyphae. Strains MF31.2, MF45.5, and FH1.1 were cultivated on MM supplemented with different concentrations of doxycycline as indicated, and cultivated at 22°C for 2 days. Hyphae were analyzed via confocal time lapse microscopy. Individual hyphae were analyzed in single stacks every 20 s for 5 min. The hyphal growth rate of at least 10 individual hyphae from two independent experiments was analyzed via ImageJ. Students *t*-test was used for significance determination as indicated.

In order to further interrogate modifications in secretion and colony macromorphology following titration of *arfA* expression, we concomitantly monitored hyphal morphology and GlaA localization (Figure 4), in addition to quantitative assessment of growth rates (Figure 5) for strains MF31.2, MF45.5 and FH1.1. Addition of 1 μg/ml doxycycline resulted in wildtype-like morphology (Figure 4) and growth rates (Figure 5) for all tested isolates. However, intracellular localization of the reporter protein glucoamylase was still observed for some hyphae of isolate MF45.5 (Figure 4).

Following addition of 0.25 μg/ml of doxycycline to growth media, isolates MF31.2 and FH1.1 demonstrated comparable growth rates, hyphal morphology, and absence of intracellular GlaA. However, loss-of-function isolate MF45.5 resulted in abnormal hyphal branching, curved growth, and swelling (Figure 4) and reduced growth rate (Figure 5). This was surprising, given that biomass was comparable between MF45.5 and the progenitor control in shake flask culture (Figure 3A). We hypothesize that the most likely explanation of the discrepancies between hyphal growth rates on solid media (Figure 5) and biomass in shake flask culture (Figure 3A) are related to the different growth parameters between the assays, which were 22°C for 28 h and 30°C for 72 h, respectively. These data suggest that ArfA regulation in *A. niger* is partially dependent on temperature, and/or a solid growth substrate.

Additionally, under loss-of-function conditions using 0.25 μg/ml, isolate MF45.5 demonstrated significant punctate localisation of intracellular GlaA, with numerous hyphae demonstrating rupture of hyphal compartments (Figure 4). This bursting was followed by release of GlaA into the surrounding medium (Figure 4). It is notable that from over 100 hyphae analyzed, cellular burst occurred mostly at intercalary regions (29%), whereas bursting of hyphae at the tip was considerably less frequent (5%, Figure 4). Hyphal bursting may contribute to decreased GlaA secretion in loss-of-function isolate MF45.5 during submerged growth due to cell death from intercalary rupture (Figure 3D).

Interestingly, when *arfA* was overexpressed using 10 μg/ml doxycycline in isolates MF45.4 and FH1.1, intracellular GlaA was not observed, and morphology was comparable to the control isolate (Figure 4). However, growth rates were impaired relative to MF31.2 for both gain-of-function strains (Figure 5). This latter observation likely explains significant changes in colony macromorphology in submerged culture (Figure 3B). Taken together, these data further support a critical role for ArfA in regulating growth, morphology, and secretion in *A. niger*.

### Overexpression of *arfA* results in increased localisation of GlaA at septa

**Figure 6:**
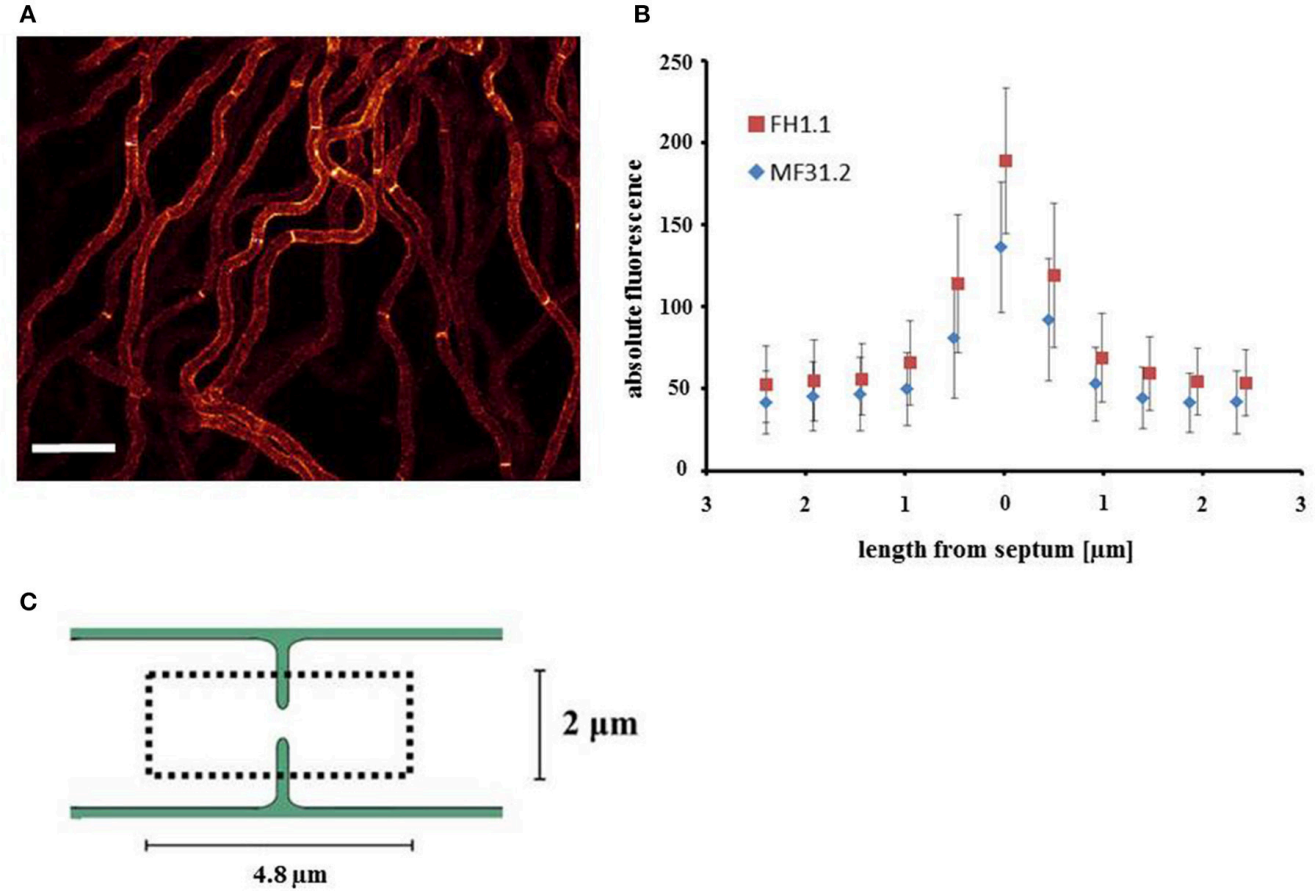
Localisation of GlaA at the hyphal septa increases following *arfA* overexpression. Strains expressing a GlaA-dtomato reporter protein, and enabling doxycycline mediated wildtype, reduced, and overexpression of *arfA* (MF31.2, MF45.5, and FH1.1, respectively), were grown at 22°C for 2 days on MM plates. After 1 h of incubation with liquid MM supplemented with respective concentrations of doxycycline, confocal microscopy of GlaA-dtomato was performed and Z-stack series were taken (dark red = low GlaA-dtomato signal, bright yellow = high GlaA intensity signal. **(A)** Exemplar fluorescent image of growing colonies demonstrated GlaA localizes to hyphal septa in the control and overexpression strain FH1.1 using 10 μg/ml doxycycline. **(B)** Septum localized GlaA-dtomato fluorescence of 12 hyphal septa from projected Z-stacks was quantified for MF31.2 and FH1.1 grown in the presence of 10 μg/ml doxycycline, which confirmed increased expression of GlaA, especially at the hyphal septum following *arfA* overexpression. Standard error bars are shown from 12 replicates. A schematic overview of the region analyzed in this analysis is depicted in **(C)**, whereby we took the septum as the central point and measured up to 2.4 μm from this point either side, giving a total length of 4.8 μm.

When conducting the above assessment of morphology and GlaA localization, an unexpected observation was the detection of GlaA-dtomato at hyphal septa (Figure 6A). Fluorescent signal was observed at septa in all three isolates (Figure 6A and Supplementary Figure S3). We consequently interrogated whether *arfA* expression levels had any impact on GlaA at these loci. Titration of doxycycline had no effect on the intensity of GlaA distribution in the control strain MF31.2. Additionally, intensity of the GlaA reporter was not impacted by *arfA* loss-of-function in isolate MF45.5 (Supplementary Figure S3). However, raising the concentration of doxycycline to 10 μg/ml increased the intensity of the GlaA at septa in both doxycycline controlled *arfA* expression strains (MF45.5 and FH1.1), suggesting that septal localisation of GlaA may be ArfA-dependent (Figures 6B,C and Supplementary Figure S3). Consequently, we measured the signal intensity at septa in isolates MF31.2 and FH1.1, which indicated fluorescent intensity of the GlaA reporter was indeed higher at septal locations following ArfA overexpression (Figure 6B). Quantification of GlaA abundance from fluorescence data indicated that approximately one third more protein was present at septa in the over-expression isolate when compared to the control strain (Table 2). Although these data do not demonstrate secretion of GlaA at hyphal septa, localisation of this protein at these loci is consistent with exocytosis of starch degrading enzymes into septal periplasmic space in the industrial fungus *A. oryzae* (Hayakawa et al., 2011). It is interesting to speculate that increased GlaA secretion in shake flask culture following ArfA overexpression (Figure 3D) could, at least in part, occur via secretion at hyphal septa.

**Table 2:** Quantitative abundance of GlaA at hyphal septum determined from fluorescent microscopy.

**Strain**	**GlaA Abundance**		
	**Adaxial**	**Abaxial**	**Total**
MF31.2	149.2	149.5	298.7
FH1.1	192.6	204.8	397.4

*Septa were taken as central points and absolute fluorescence was measured over a length of 2.4 μm in either adaxial or abaxial direction (Figures 6B,C). GlaA concentration was approximated based on polynomial function of the 6th order. Integrals of these functions were determined over the length of 2.4 μm. These values were used as proxy measures for GlaA abundance, indicating that the arfA over-expression isolate had approximately a third more GlaA at hyphal septa when compared to MF31.2 control*.

### Depletion and overexpression of *arfA* affects actin ring positioning

**Figure 7:**
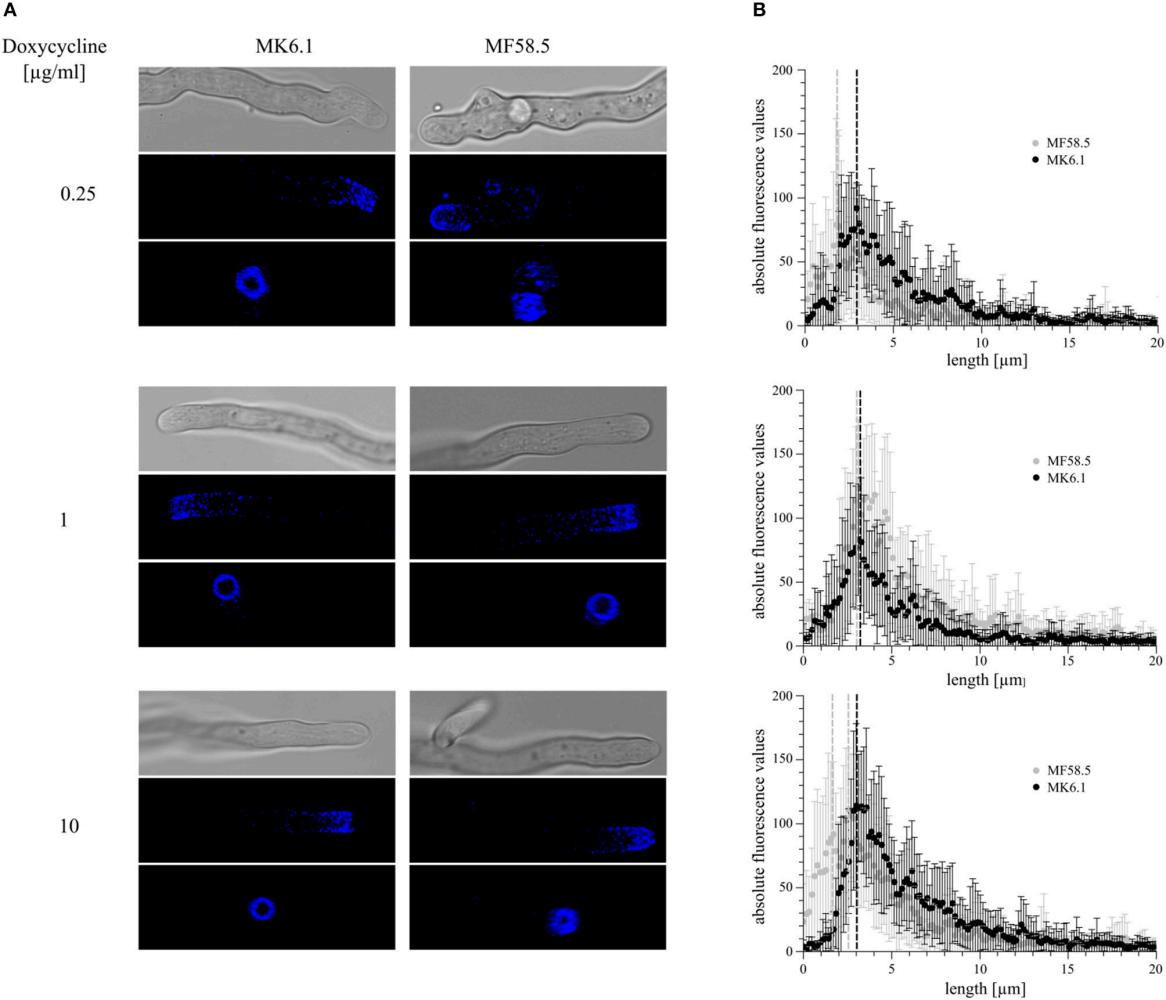
Modifications to *arfA* expression shift the actin ring toward the hyphal apex. In order to assess the location of the endocytic actin ring, strains MF58.8 and MK6.1 were utilized, which both express the actin binding protein AbpA tagged with cyan fluorescent protein (AbpA::CFP). Isolate MK6.1 was used as control to assess the actin ring position under native *arfA* expression. Strain MF58.5 has the native *arfA* deleted, and titratable *arfA* expression using the Tet-on system. Isolates were cultivated at 22°C for 2 days on MM supplemented with various concentrations of doxycycline and analyzed via confocal microscopy. Z-stacks of 15 individual hyphal tips were taken **(A)**, and representative pictures of DIC (upper panels) and projected 3D images of Z-stacks (middle and bottom panels) are shown. These data indicated structural disruption of the actin ring following reduced/increased *arfA* expression, and a clear shift of AbpA::CFP toward the hyphal apex. Quantification of AbpA::CFP fluorescence along the cell membrane 20 μm from the tip apex **(B)** revealed a shift in actin ring position in ArfA loss- and gain-of-function mutants. Fifteen hyphae/strain/condition were assessed, with bars depicting standard error. Dotted lines denote the predicted position of the actin ring. Note two possible positions are shown for MF58.5 under 10 μg/ml dox due to deterioration of ring structure observed in **(A)**.

In order to ascertain whether titratable expression of *arfA* impacts the cytoskeletal apparatus, we constructed a strain in which expression of *arfA* was controlled via the Tet-on system and where the actin binding protein AbpA was fluorescently labeled with CFP (MF58.5). The previously published strain MK6.1 (Kwon et al., 2013) expressing AbpA::CFP with no modifications in *arfA* gene expression served as respective control (Table 1). The actin ring consists of actin, myosin II, and associated proteins. In the wildtype background (strain MK6.1), this ring is localized about 3 μm behind the tip (Figure 7). When both strains were grown on 1 μg/ml doxycycline, the localization of the actin ring was unaltered, although fluorescence was slightly increased in MF58.5 (Figure 7B). The amount of AbpA decreased when ArfA was limited via addition of 0.25 μg/ml doxycycline. Interestingly, the actin ring was shifted approximately 1.2 μm toward the tip (Figure 7B). When the concentration was raised to 10 μg/ml doxycycline, higher fluorescence levels compared to the wildtype was observed, and the actin ring shifted again toward the tip (0.6 μm) and was more spread toward the apical dome (Figure 7B). Quantitative analyses of fluorescent intensity at either 3 or 2 μm confirmed this actin ring shift toward the hyphal tip in *arfA* conditional expression mutants (Supplementary Figure S4). From the obtained images we measured and calculated the base area of the apical dome, showing that the area was reduced during downregulation and overexpression of *arfA* (Table 3). These data indicate that titration of *arfA* expression impacts the *A. niger* cytoskeleton in addition to the secretory pathway.

**Table 3:** *arfA* expression influences the base area of the apical dome.

**Strain**	**Doxycycline [**μ**g/ml]**		
	**0.25**	**1**	**10**
MK6.1	**35.69** ± **2.61**	**35.88** ± **4.36**	**35.90** ± **4.98**
	*100 ± 7.32*	*100 ± 12.17*	*100 ± 13.87*
MF58.5	**23.96** ± **4.30**	**37.19** ± **6.73**	**27.76** ± **2.93**
	*67.15 ± 12.05*	*103 ± 18.76*	*77.31 ± 8.18*

*Calculation was done using values from eight individual hyphae of MF58.5 and MK6.1, which were used for the analysis of actin ring position in Figure 6. Area is given in [μm^2^] (bold) and respective percentage corresponding to MK6.1 (italics)*.

### ArfA does not localize with the ER or at the hyphal tip in *A. niger*

**Figure 8:**
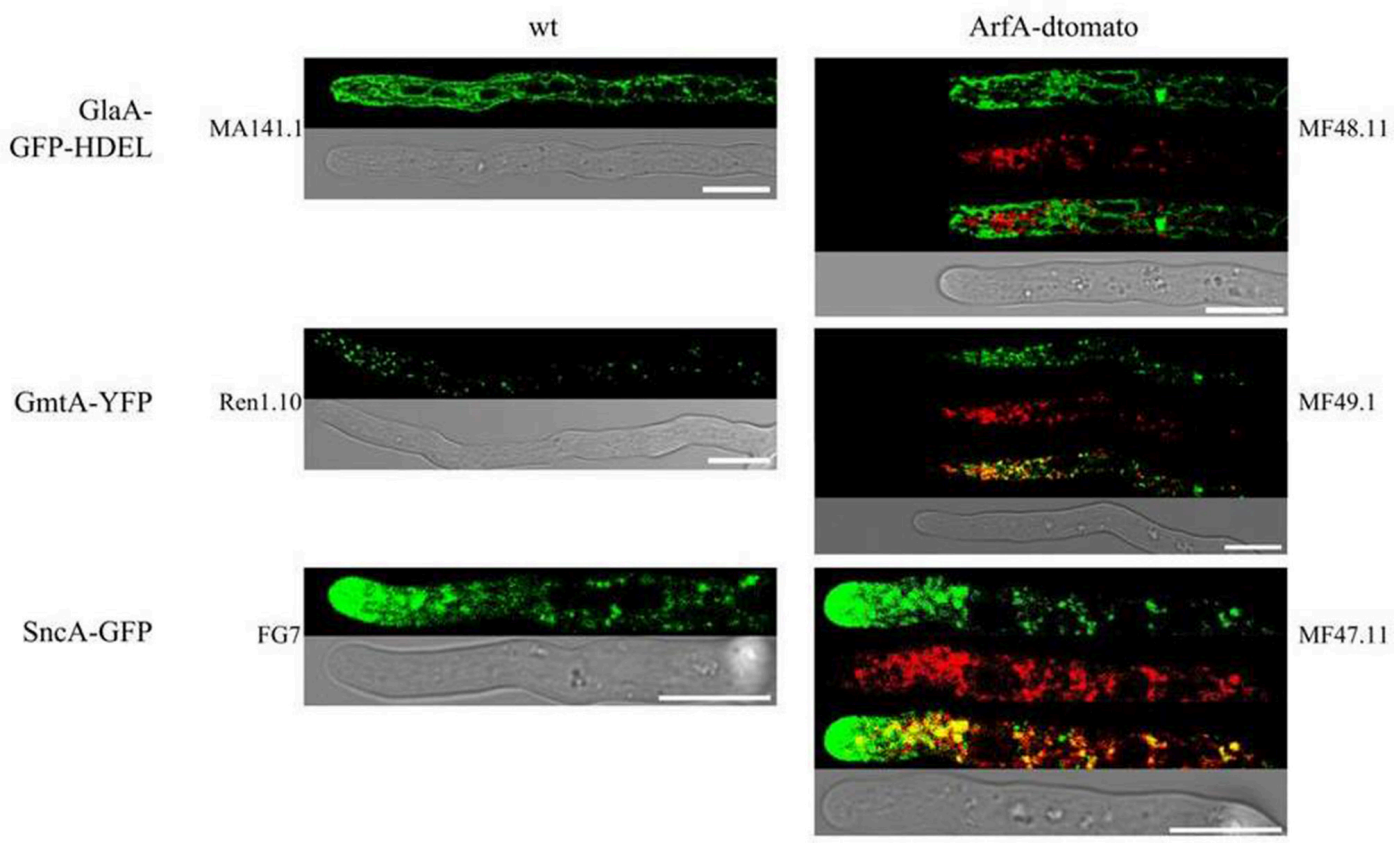
ArfA does not localize with the ER or at the hyphal tip in *A. niger*. Strains were cultivated on MM agar plates at 22°C for 2 days. Co-localization studies utilized strains which each have a specific component of the secretory system tagged, including the ER (GlaA-GFP-HDEL, isolate MA141.1), Golgi (GmtA-YFP, isolate Ren1.10) and post-Golgi carriers (SncA-GFP, isolate FG7). Localization of each reporter demonstrated expected localization of each secretory component (left panels). Co-localization experiments were conducted by expressing an ArfA-dtomato reporter in isolates MF48.11, MF49.1, and MF47.11, respectively, which revealed ArfA does not localize to the ER or the hyphal tip. Partial co-localization was observed between ArfA and Golgi and Golgi carriers. Scale bars on all images represent 10 μm.

Arf GTPases can localize to a multiple subcellular loci, for example specific regions of the Golgi (Stearns et al., 1991; Lee and Shaw, 2008), and dynamic spots of the plasma membrane (Donaldson, 2003; Smaczynska-de Rooij et al., 2008). In order to establish the subcellular localisation of ArfA, we generated various isolates with fluorescently labeled components of the secretory system. Additionally, the endogenous *arfA* gene was replaced with a C-terminally dtomato tagged *arfA* gene. C-terminal tagging was chosen, because it has been demonstrated in *A. nidulans* that N-terminal myristoylation is crucial for correct localization of ArfA (Lee and Shaw, 2008). ArfA tagging was conducted in previously published *A. niger* reporter strains, in which distinct steps of the secretory pathway have been fluorescently labeled (Table 1 and Figure 8): (i) the ER (GlaA-GFP-HDEL), (ii) Golgi (GmtA-YFP), and (iii) post-Golgi carriers in which the v-SNARE SncA is fluorescently tagged (SncA-GFP) (Carvalho et al., 2011; Kwon et al., 2011). Additionally, a strain carrying only the ArfA-dtomato expression construct (MF56.7) was constructed, to exclude the possibility that combined expression of two fluorescently labeled proteins of the secretory pathway interferes with the localisation of ArfA. All strains were viable and produced spores which were able to grow on selective medium (data not shown), and did not display any defects in standard growth assays using solid media (data not shown), suggesting that replacing the essential *arfA* with the *arfA-dtomato* led to functional ArfA-dtomato fusion protein.

Subcellular localisation analyses of ArfA reporter strains showed a punctuate localization of this protein within growing hyphae (Figure 8). ArfA is not distributed to the hyphal tip (Figure 8), an observation supported by linescan analyses along the hyphal length (Supplementary Figure S5). As shown in Figure 8, ArfA does not co-localize with the ER marker. We noted partial co-localization of ArfA with Golgi associated GmtA and with post Golgi carries as determined by SncA localisation (Figure 8). However, in both these latter experiments, ArfA did not exclusively localize with either marker. These data were surprising, given that the ArfA orthologue Arf1 has been demonstrated to predominantly localize at the Golgi in a diverse range of organisms, including mammals (Stearns et al., 1991), *S. cerevisiae* (Donaldson and Klausner, 1994; Suda et al., 2018), and *A. nidulans* (Lee and Shaw, 2008). Our data do not conclusively confirm the sub-cellular localization of ArfA in *A. niger*, but are suggestive of association at an intercalary hyphal region that is also distinct from the ER. With regards to the observed changes in actin ring position in *A. niger* (Figure 7), Arf3 in yeast, and Arf6 in mammals regulate actin organization by activating GAPs at the plasma membrane (Donaldson, 2003; Smaczynska-de Rooij et al., 2008; Hsu and Lee, 2013). Our localization experiments suggest that this is unlikely to be the case for ArfA in *A. niger*. We therefore hypothesize that modifications to actin ring position (Figure 7) occur as a consequence of defective secretion following titration of *arfA* expression.

## Discussion

In this study, we have functionally characterized the *A. niger* ArfA. The ArfA orthologue, Arf1, has been most extensively studied in the budding yeast *S. cerevisiae*, where this protein controls the formation of vesicle coats at distinct steps in intracellular transport. Arf1 in the GDP-bound form is located in the cytosol and is activated by GEFs Gea1/2 or Sec7, which are located at cis-cisternae and trans-cisternae of the Golgi, respectively (Wright et al., 2014). This positional distribution of Arf1-GEFs enables differential effector recruitment by GTP-bound Arf1, specifically the COPI vesicles at cis-cisternae, which are required for recycling of Golgi glycoslylation enzymes, and clathrin-coated vesicles at trans-cisternae, which facilitate carrier formation (Suda et al., 2018). Such control of vesicle trafficking is essential in yeast, as double deletion of *ARF1/2* is lethal (Stearns et al., 1990). Data generated in this study suggests that ArfA may perform a comparable regulatory function in *A. niger*. Firstly, a complementation approach using *S. cerevisiae* confirmed that this protein is highly likely to function as an ADP–ribosylation factor that can regulate vesicle trafficking in yeast. Next, by putting the *arfA* gene under control of the Tet-on system in *A. niger*, we were able to concomitantly mimic and analyse downregulation, overexpression and wildtype phenotypes. These loss-of-function analyses demonstrated this gene is also essential in *A. niger*. Subsequent phenotypic screens of *A. niger arfA* conditional expression isolates suggested that the ArfA protein plays a critical regulatory role in secretion, as loss- and gain-of-function isolates were sensitive to various secretory stressors. These data are consistent with defective vesicle trafficking and secretion in *ARF1* mutants of different fungi such as *S. cerevisiae, C. albicans, A. nidulans*, and *M. circinelloides* (Stearns et al., 1991; Lee and Shaw, 2008; Labbaoui et al., 2017; Patiño-Medina et al., 2017). Interestingly, there are deviations in the relative importance of Arfs among Ascomycetes. For example, in contrast to this study, functional analyses of Arf1-3 in *C. albicans* demonstrated that Arf2, but not Arf1, was essential for cell viability (Labbaoui et al., 2017). It is plausible that these discrepancies can be related to subtle differences in Arf function between species, or the disparate lifestyles or environmental niches in which they inhabit.

Interestingly, titratable expression of *arfA* resulted in numerous effects during *A. niger* growth in submerged culture, with colony macromorphology, total protein secretion, and extracellular titres of glucoamylase variously impacted. Increased *arfA* expression led to reduced pellet diameter, a dispersed hyphal morphology, and increased total protein secretion. Elevated titres of secreted protein in an over-expression isolate that concomitantly demonstrates sensitivity to secretory stress was surprising, and may be due to modifications in colony macromorphology under these conditions. For example, in filamentous fungal cell factories, increased protein secretion often correlates with a dispersed morphology as the number of hyphal tips/biomass increases. Numerous techniques, including generation of temperature sensitive mutants (Kwon et al., 2013), and addition of inorganic insoluble micro particles (Wucherpfennig et al., 2012), have been developed to achieve this colony macromorphology in submerged culture. We hypothesize that careful titration of *arfA* expression may enable optimized morphology in submerged culture, which ultimately may provide novel avenues for improved titres of total secreted protein.

In contrast to over-expression strains, loss-of-function mutants displayed comparable pellet diameter and total secreted protein, yet significantly reduced glucoamylase secretion during submerged culture. Microscopic examination provided two possible explanations for these observations. Firstly, fluorescently labeled GlaA remained intracellular, which is consistent with defective secretion following reduction of ArfA levels. Secondly, reduced glucoamylase secretion may also be attributed to loss of cell viability following intercalary hyphal rupturing.

These data highlight the complex interplay between defects in secretion, aberrant growth rates, morphological changes, and colony macromorphology following titration of *arfA* expression. Consequently, increased characterization of Arfs, and the phenotypic consequences of their mis-expression in other filamentous fungi, will complement the extensive work already conducted in *S. cerevisiae* and mammals.

**Figure 9:**
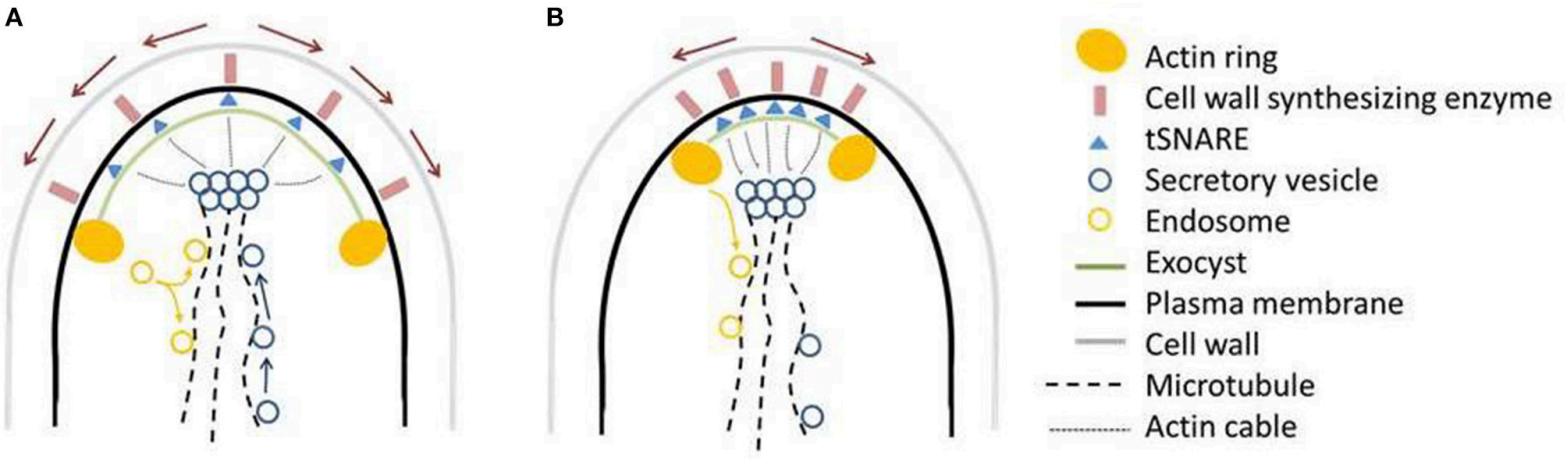
Working model for ArfA dependent secretion, actin ring positioning, hyphal growth, and morphology**. (A)** In wildtype hyphal tips, secretory vesicles containing cell end markers, wall synthesizing enzymes and other cargo move along microtubules and actin cables to the Spitzenkörper, and subsequently are fused to the plasma membrane by the exocyst/SNARE proteins, releasing hydrolytic/cell wall synthesizing enzymes. The actin ring is fixed a constant position of about 3 μm from the apex. Growth of the plasma membrane moves SNAREs/cell wall synthesizing enzymes to the posterior of the hypha (red arrows), which become endocytosed at the actin ring. Endosomes either recycle the contents back to the plasma membrane, presumably via the Spitzenkörper, or back to the anterior of the cell along microtubules as shown for *A. nidulans* (Taheri-Talesh et al., 2008). **(B)** Following a reduction or increase in ArfA levels, the secretory pathway is defective, disrupting supply of markers, enzymes, and other cargo. Either due to direct ArfA regulation, or due to this disruption of secretory cargo, the actin ring shifts an average of 0.6–1.2 μm toward the hyphal tip. Both defective vesicle formation, and actin ring position, may synergistically contribute to the pleiotropic phenotypic consequences of *arfA* mis-expression. For further discussion see main text.

Intriguingly, fluorescent microscopy demonstrated that the position of the actin ring at the hyphal tip is modified following titratable *arfA* expression in *A. niger* (Figure 7). Arfs are known to play important regulatory roles in actin dynamics in a range of organisms. For example, in *S. cerevisiae*, Arf3 is required for organization of actin cables, cortical patches and endocytosis (Lambert et al., 2007). Arf3 regulates the GTPase activating protein Bud2p during axial budding (Hsu and Lee, 2013), which is a component of a module with the GTPase Bud1, and the GTP/GDP exchange factor Bud5. This module activates Cdc24, which subsequently activates Cdc42, which in turn regulates actin dynamics necessary for polar growth (Hsu and Lee, 2013). The Arf3 orthologue in mammals, Arf6, also plays a regulatory role in actin polymerization (D'Souza-Schorey et al., 1997). Data presented in this study demonstrates that under native *arfA* expression levels, the actin ring is positioned about 3 μm behind the hyphal apex, but moves toward the tip when *arfA* is downregulated (about 1.2 μm) or upregulated (about 0.6 μm). It is possible that the shift in actin ring position following titration of *arfA* expression occurs due to defective delivery of necessary molecular markers to the tip. Indeed, in yeast, the position of actin patches is dependent on lipid domains (Cortesio et al., 2015), and the composition of these signaling lipids might be impacted by modifications in secretory pathway following *arfA* mis-expression. Alternatively, as is the case for Arf3 in *S. cerevisiae* and Arf6 in mammals, there might be a direct interaction of ArfA with regulatory modules that are necessary for actin organization in *A. niger*. However, given that Arf3 and Arf6 localize to the plasma membrane (Donaldson, 2003; Smaczynska-de Rooij et al., 2008), and ArfA in this study localized to intercalary region of growing hyphae (Figure 8), we hypothesize that modifications in actin position are due to defective trafficking of lipid domains or membrane and cell wall components (Figure 9).

Nevertheless, our study does suggest that changes in actin ring position could partially account for the phenotypic consequences of *arfA* mis-expression. The actin ring is the site of endocytosis, and the location of this cytoskeletal apparatus at the hyphal tip is vital for polar growth (Taheri-Talesh et al., 2008). In *A. nidulans*, the actin ring is maintained precisely 1–2 μm behind the hyphal apex, even in rapidly growing hyphae (Taheri-Talesh et al., 2008). This spatial maintenance is likely mediated by actin cables, and occurs despite the short lifespan of actin patches from which the ring is composed [<1 min (Taheri-Talesh et al., 2008)]. Such spatial control of the actin ring enables recycling of exocytic vesicle membrane and v-SNAREs and cell wall synthesizing enzymes as they move to posterior regions of the hyphae due to plasma membrane growth. This removal from posterior regions of the hyphae ensures markers for vesicle fusion are located exclusively at the apex, and stops plasma membrane expansion due to secretory vesicle membranes (Taheri-Talesh et al., 2008; Takeshita et al., 2013). Further importance of the tightly maintained subcellular location of the actin ring in fungal hyphae has been predicted by mathematical growth models using *C. albicans*, which have subsequently been verified using quantitative measurements of fluorescently labeled components of the exocytic and endocytic machinery (Caballero-Lima et al., 2013). Consequently, the spatial control of the actin ring behind the hyphal apex (1–2 μm) in *A. nidulans* (Taheri-Talesh et al., 2008), and 3 μm in *A. niger* (Figure 7) may ensure that endocytosis and recycling of cell wall synthesizing enzymes occurs at a precise location that maintains polarized growth while avoiding hyphal swelling. We hypothesize that the observed defects in growth rates and morphology in *arfA* loss- and gain-of-function mutants can be firstly attributed to defects in secretion, which may either cause, and/or be compounded by, modulation of actin ring position (Figure 9).

This study also suggests that the *A. niger* glucoamylase might not only be secreted at the hyphal tip, but also at hyphal septa as previously suspected (Gordon et al., 2000). This is consistent with studies using *A. oryzae*, where the major extracellular protein alpha-amylase was observed to localize in the space between the plasma membrane and cell wall at septa [i.e., the septal periplasm (Hayakawa et al., 2011)]. Septal exocytosis is required for secondary cell wall thickening, intercalary hyphal growth, and branch initiation in filamentous fungi (Hayakawa et al., 2011; Read, 2011). Interestingly, microscopic monitoring of fluorescent dye uptake has demonstrated that exocytosis is also coupled with endocytosis at intercalary loci in a variety of fungi, including *A. nidulans* (Fischer-Parton et al., 2000). In our study, overexpression of *arfA* resulted in increased septal GlaA localisation. It is thus possible that ArfA is also required for normal exocytic and/or endocytic processes at the hyphal septum.

In conclusion, we have functionally characterized the essential protein ArfA in the filamentous cell factory *A. niger*. ArfA has critical implications for fungal growth rates, and hyphal/colony morphology. Moreover, by titrating *arfA* expression at different levels, we were able to increase the amount of secreted proteins. Actin ring position was also impacted by *arfA* expression levels, which might impact plasma membrane and cell wall growth at the hyphal apex. Finally, our data suggest that septal secretion may occur in *A. niger*. Further studies are required to determine if secretion at septa does indeed occur in *A. niger*, and whether this process could be harnessed in industrial applications for improved titres of useful proteins.

## Availability of data and materials

Data and materials generated during and/or analyzed in the current study are available from the corresponding author on request.

## Author contributions

VM and MF conceived of the study; MF, OK, and CK conducted experiments; VM, MF, and TC analyzed and interpreted data, generated figures, and wrote the manuscript; Lars Barthel is acknowledged for helping with the statistical analyses. All authors read and approved the final manuscript.

### Conflict of interest statement

The authors declare that the research was conducted in the absence of any commercial or financial relationships that could be construed as a potential conflict of interest.
