# Supplementary material for: Conditional Expression of the Small GTPase ArfA Impacts Secretion, Morphology, Growth, and Actin Ring Position in Aspergillus niger
Source: Front Microbiol. 2018 May 8;9:878. doi: 10.3389/fmicb.2018.00878 (PMC5952172; doi:10.3389/fmicb.2018.00878)
Supplement: Supplementary file 1 [file Table_1.docx]

**Supplemental Table S1: Primers used in this study**

| **Construct** | **Primer** | **Sequence (5’ to 3’ oriented)** | **Target** | **ORF code** |
| --- | --- | --- | --- | --- |
| pMF30.1 | GlaA_gpdA_fw | ccgcttgagcagacatcaccgaattcATGTCGTTCCGATCTCTACTCG | *glaA* | An03g06550 |
|  | GlaA_fu_rev | caccggctccagcgcctgcaccagctccGGTCGAGGTCACGCTGC |  |  |
|  | dtomato_fu_fw | ggagctggtgcaggcgctggagccggtgcaATGGTGAGCAAGGGCGAGG | *dtomato* |  |
|  | dtomato_fu_rev | aagtggatcccggtcggcatctactgtttaaacTTACTTGTACAGCTCGTCCATGC |  |  |
| pFH1.3 | ArfA_express_fw | cttgagcagacatcaccgtttaccATGGGTCTCACTTTCTCCAAGT | *arfA* | An08g03690 |
|  | ArfA_expres_rev | ccggtcggcatctactgtttTTAGTCGCGGTTCGTTTTCC |  |  |
| pCK1.1 | ParfA_fw | caccatatccatccggatggctcgagCACACGAGAGCCTCCGTTTA | *5' arfA* | An08g03690 |
|  | ParfA_hyg_rev | caattccagcagcggcttGAGAGTCCAGGATGTGCGAG |  |  |
|  | hygP6f | aagccgctgctggaattgGGCTCTGAGGTGCAGTGGAT | *hygR* |  |
|  | pJet+hygP9r | ttgtaggagatcttctagaaagatGGCGTCGGTTTCCACTATC |  |  |
| pCK2.6 | TarfA_hyg_fw | acacggcacaattatccatcgCTGGTGACGGTCTGTACGAG | *3' arfA* | An08g03690 |
|  | arfabiparnew | ttgtaggagatcttctagaaagatTATTTGAGGACCGTGCCGAC |  |  |
|  | hygbipartitefw | ctcgagtttttcagcaagatAAAGTTCGACAGCGTCTCC | *hygR* |  |
|  | hygP7r | cgatggataattgtgccgtgTTGGGTGTTACGGAGCATTCA |  |  |
| pMF44.6 | ArfA_fluo_fw | atccatccggatggctcgagATCTCGCACATCCTGGACTC | *arfA* | An08g03690 |
|  | ArfA_fluo_rev | tgcaccggctccagcgcctgcaccagctccGTCGCGGTTCGTTTTCC |  |  |
|  | dtomato_fu_fw | ggagctggtgcaggcgctggagccggtgcaATGGTGAGCAAGGGCGAGG | *dtomato* |  |
|  | dtomato_trpC_rv | ctcagagccCGCGTGGAGCCAAGAGC |  |  |
|  | hyg_fu_fw | ctccacgcgGGCTCTGAGGTGCAGT | *hygR* |  |
|  | hyg_Nde_rev | GATACACATGGGGATCAG |  |  |
| pMF48.1 | ArfAyeastexp_fw | agaacttagtttcgacggatccATGGGTCTCACTTTCTCCAAGT | *arfA* | An08g03690 |
|  | Arfyestexp_rv | cttatctaggtcgacggatccTTAGTCGCGGTTCGTTTTCC |  |  |
| pMF46.3 | ARF1_express_fw | ggtatcgataagcttgatatcgaattcTGCACAGTTGAATTCTCGTCT | ARF1 | YDL192W |
|  | ARF1_express_rv | gccgctctagaactagtggatccAGGCTCTTTCTTGCACTAGGT |  |  |
| pMF49.1 | ARF1yeastexp_fw | agaacttagtttcgacggatccATGGGTTTGTTTGCCTCTAAGT |  |  |
|  | ARF1yeastexp_rv | cttatctaggtcgacggatccTTAAGTTGAGTTTTTCAAACTGTTACTTAACC |  |  |
|  | ARF2_del_fw | ttcatagagaaaagatgggtctatacgACACAGTTTTCATGAGGATGGC | MET15 | YLR303W |
|  | ARF2_del_rv | catgactaaacgttctatacagatttagaCTTGTGAGAGAAAGTAGGTTTATACATAA |  |  |

Uppercase indicates the primer binding sequence
