# Supplementary material for: Conditional Expression of the Small GTPase ArfA Impacts Secretion, Morphology, Growth, and Actin Ring Position in Aspergillus niger
Source: Front Microbiol. 2018 May 8;9:878. doi: 10.3389/fmicb.2018.00878 (PMC5952172; doi:10.3389/fmicb.2018.00878)
Supplement: Supplementary file 3 [file Presentation_1.PDF]

Conditional expression of the small GTPase ArfA impacts secretion, morphology, growth, and actin ring position in *Aspergillus niger*

Markus RM Fiedler, Timothy C. Cairns, Oliver Koch, Christin Kubisch, Vera Meyer

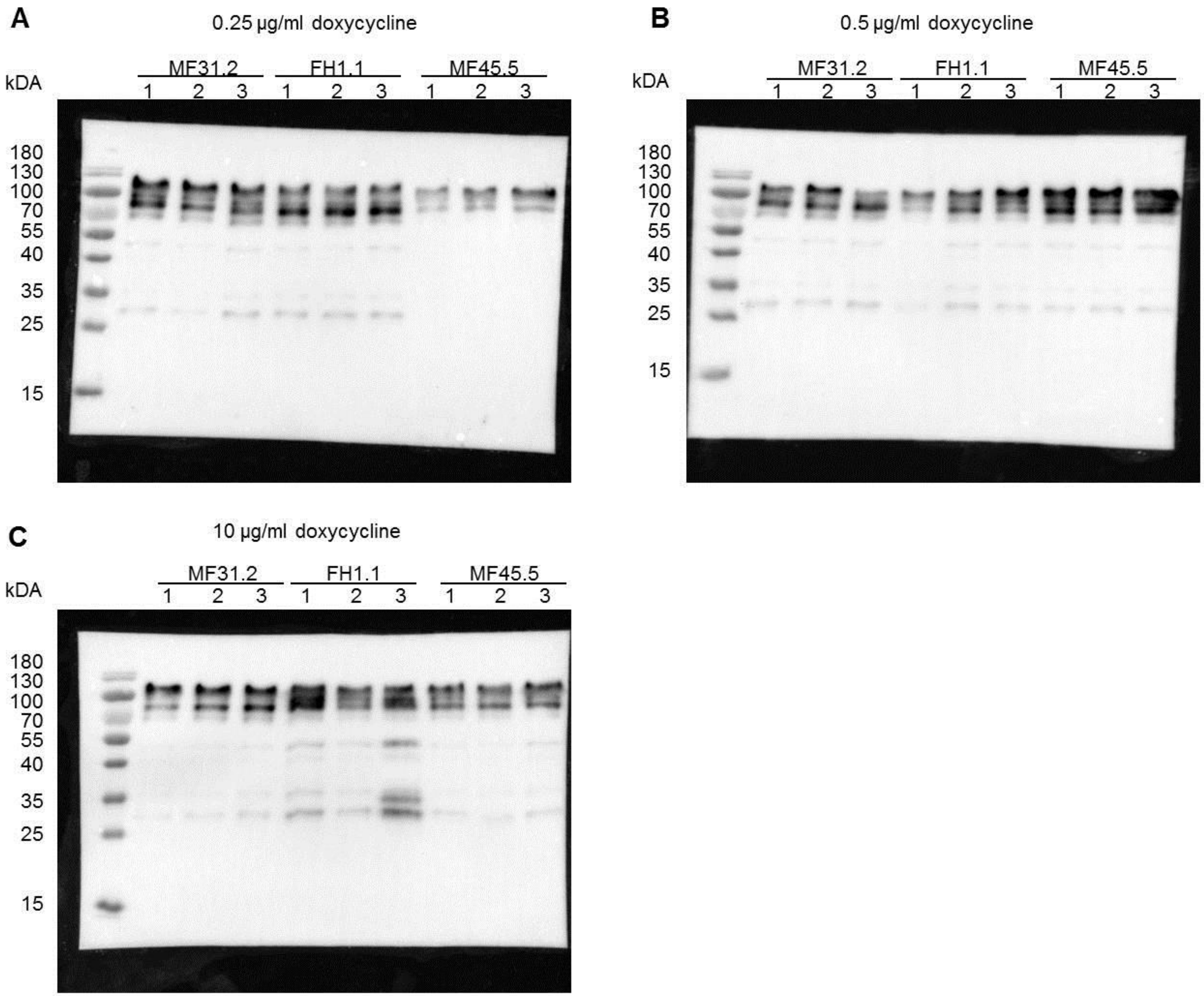

Supplemental Figure S1: Glucoamylase Western Blots of triplicate cultivations strains MF31.2, MF45.5 and FH1.1 in submerged culture. MM was supplemented with (A) 0.25, (B) 0.5 or (C) 10 µg/ml doxycycline. $5x10^6$ conidia/ml were inoculated and incubated at 30°C, 250 rpm for 72h. Equal amounts of culture supernatants were loaded onto SDS-PAGE and decorated and detected as described in Materials and Methods.

Conditional expression of the small GTPase ArfA impacts secretion, morphology, growth, and actin ring position in *Aspergillus niger*

Markus RM Fiedler, Timothy C. Cairns, Oliver Koch, Christin Kubisch, Vera Meyer

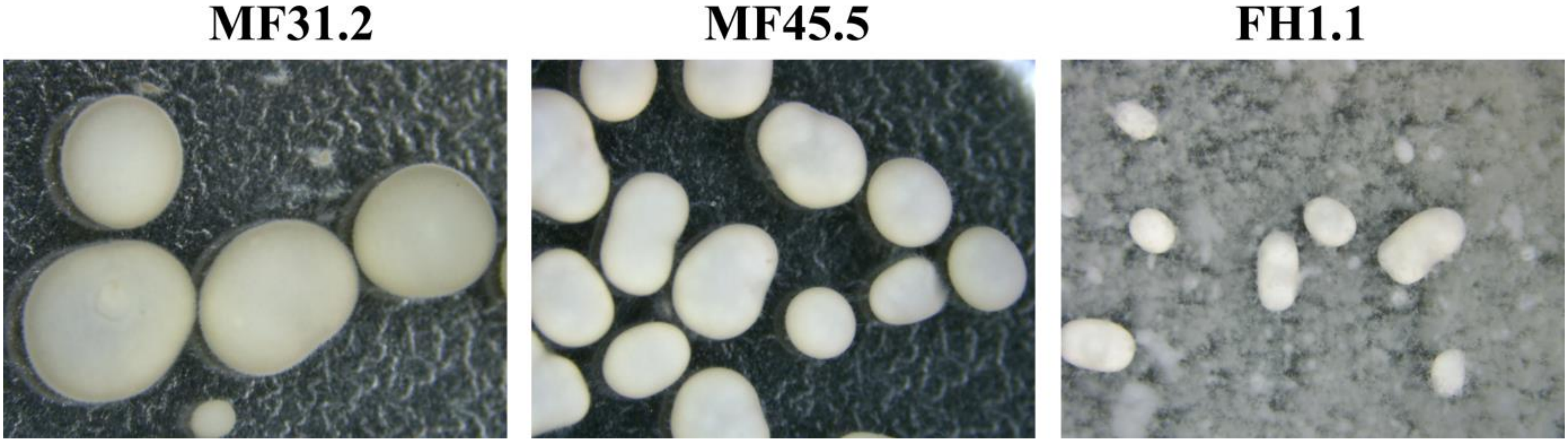

Supplemental Figure S2: Pellet morphology of strains MF31.2, MF45.5 and FH1.1 in submerged culture. MM was supplemented with 10 µg/ml doxycycline. $5x10^6$ conidia/ml were inoculated in liquid MM supplemented with 10 µg/ml doxycycline and incubated at 30°C, 250 rpm for 72h. 1 ml samples were taken and analysed via stereomicroscopy.

Conditional expression of the small GTPase ArfA impacts secretion, morphology, growth, and actin ring position in *Aspergillus niger*

Markus RM Fiedler, Timothy C. Cairns, Oliver Koch, Christin Kubisch, Vera Meyer

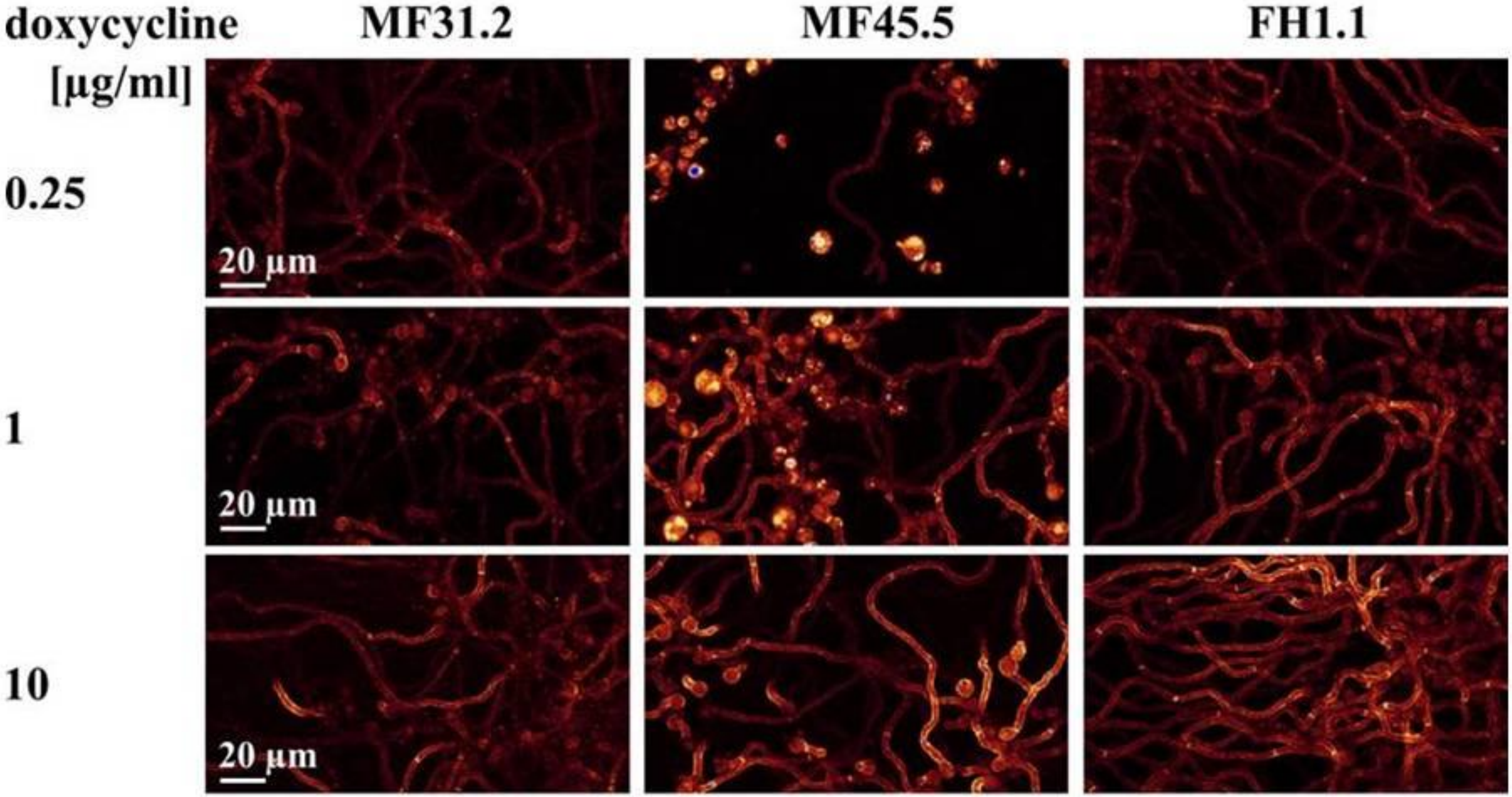

Supplemental Figure S3: Localisation of GlaA at the hyphal septa in ArfA overexpression mutants. Strains expressing a GlaA-dtomato reporter protein, and enabling doxycycline mediated wildtype, reduced, and overexpression of ArfA (MF31.2, MF45.5 and FH1.1, respectively), were grown at 22°C for 2 days on MM plates. After 1h of incubation with liquid MM supplemented with respective concentrations of doxycycline, confocal microscopy of GlaA-dtomato was performed and Z-stack series were taken (dark red= low GlaA-dtomato signal, bright yellow= high GlaA intensity signal, blue=saturated GlaA intensity signal). GlaA localises to hyphal septa in the control and overexpression strains MF31.2 and FH1.1 using 10 µg/ml doxycycline.

Conditional expression of the small GTPase ArfA impacts secretion, morphology, growth, and actin ring position in *Aspergillus niger*

Markus RM Fiedler, Timothy C. Cairns, Oliver Koch, Christin Kubisch, Vera Meyer

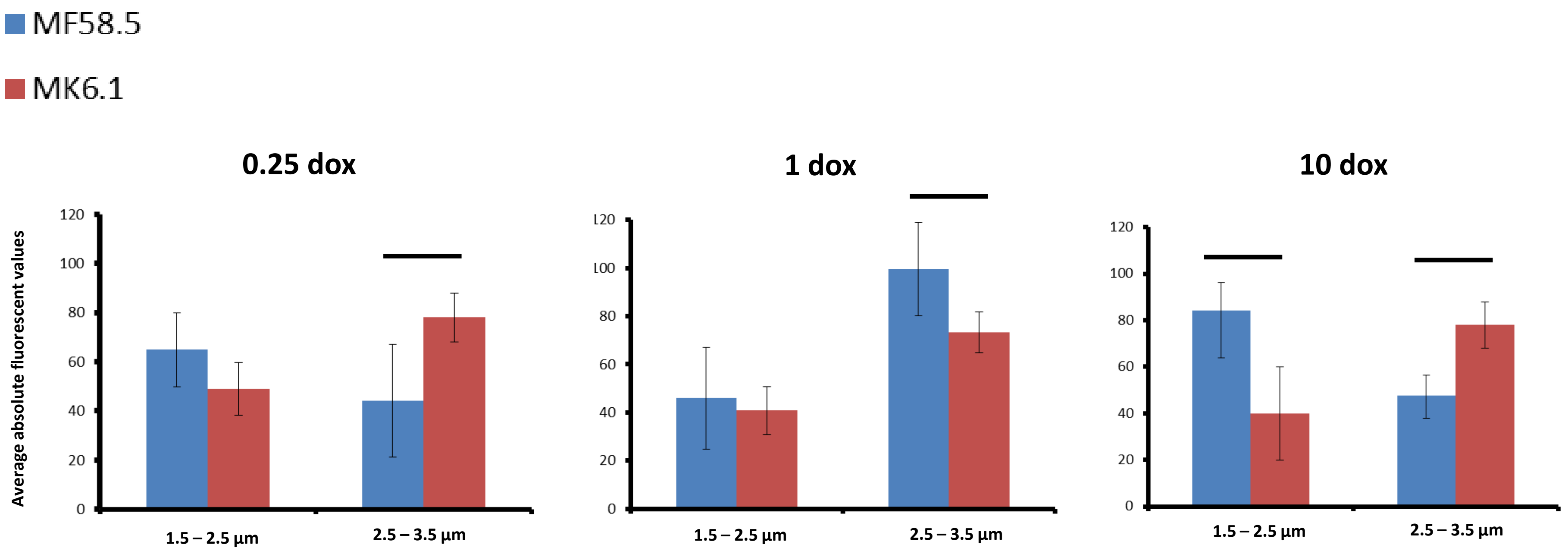

Supplemental Figure S4: Quantitative analysis of AbpA fluorescent intensity at the hyphal tip. Isolate MF58.5 has titratable control of *arfA* expression via the Tet-on system, and actin binding protein AbpA fluorescently labelled with CFP. The previously published strain MK6.1 (Kwon et al., 2013) expressing AbpA::CFP with no modifications in *arfA* gene expression served as respective control. We measured AbpA fluorescent intensity at either 2 or 3 µm (+/- 0.5 µm) for 15 hyphae for in both strains, and at either 0.25, 1 or 10 µg/ml dox concentrations. Vertical bars are standard error amongst samples. We found a clear shift in AbpA fluorescent intensity towards the hyphal tip in isolate MF58.5 at both 0.25, or 10 µg/ml dox concentrations when compared with MK6.1 control. Horizontal bars indicate statistically significant differences ($p < 0.05$) in students t-test.

Conditional expression of the small GTPase ArfA impacts secretion, morphology, growth, and actin ring position in *Aspergillus niger*

Markus RM Fiedler, Timothy C. Cairns, Oliver Koch, Christin Kubisch, Vera Meyer

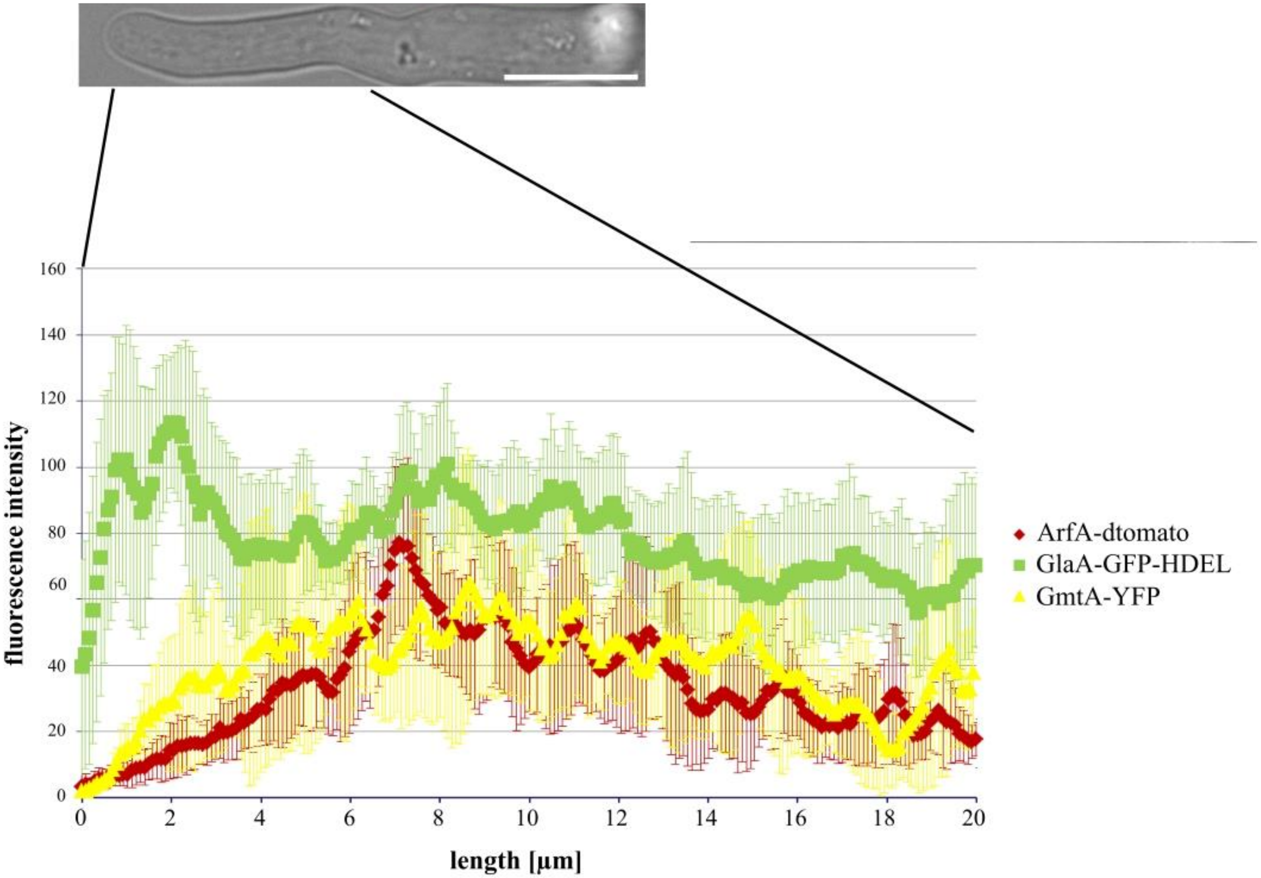

Supplementary Figure S5: Line scan analysis of reporter intensity from the hyphal tip Fluorescent intensity along hyphae was quantified for ArfA, ER and Golgi fluorescent signals. This demonstrated a clear lack of ArfA at the hyphal tip. Mean fluorescence expression values along z-stacks of 20 hyphae 20 µm from the hyphal tip are shown (C). Scale bars on images represent 10 µm.
